# Bone morphogenic protein-4 availability in the cardiac microenvironment controls inflammation and fibrosis in autoimmune myocarditis

**DOI:** 10.1038/s44161-024-00432-0

**Published:** 2024-02-19

**Authors:** Christian Perez-Shibayama, Cristina Gil-Cruz, Nadine Cadosch, Mechthild Lütge, Hung-Wei Cheng, Angelina De Martin, Kira Frischmann, Anna Joachimbauer, Lucas Onder, Iliana Papadopoulou, Chrysa Papadopoulou, Sandra Ring, Philippe Krebs, Vivian P. Vu, Matthias P. Nägele, Valentina A. Rossi, Danaë Parianos, Valentin W. Zsilavecz, Leslie T. Cooper, Andreas Flammer, Frank Ruschitzka, Peter P. Rainer, Dörthe Schmidt, Burkhard Ludewig

**Affiliations:** 1 Institute of Immunobiology, Kantonsspital St. Gallen, St. Gallen, Switzerland; 2 University Heart Center, University Hospital Zurich and University of Zurich, Zurich, Switzerland; 3 Institute of Pathology, University of Bern, Bern, Switzerland; 4 Graduate School for Cellular and Biomedical Sciences, University of Bern, Bern, Switzerland; 5 Division of Cardiology, Medical University of Graz, Graz, Austria; 6 Department of Cardiovascular Medicine, Mayo Clinic, Jacksonville, FL USA; 7 BioTechMed Graz, Graz, Austria; 8 St. Johann in Tirol General Hospital, St. Johann in Tirol, Austria

**Keywords:** Cardiovascular biology, T cells, Cardiac hypertrophy

## Abstract

Myocarditis is an inflammatory heart disease that leads to loss of cardiomyocytes and frequently precipitates fibrotic remodeling of the myocardium, culminating in heart failure. However, the molecular mechanisms underlying immune cell control and maintenance of tissue integrity in the inflamed cardiac microenvironment remain elusive. In this study, we found that bone morphogenic protein-4 (BMP4) gradients maintain cardiac tissue homeostasis by single-cell transcriptomics analyses of inflamed murine and human myocardial tissues. Cardiac BMP pathway dysregulation was reflected by reduced BMP4 serum concentration in patients with myocarditis. Restoration of BMP signaling by antibody-mediated neutralization of the BMP inhibitors gremlin-1 and gremlin-2 ameliorated T cell-induced myocardial inflammation in mice. Moreover, progression to inflammatory cardiomyopathy was blocked through the reduction of fibrotic remodeling and preservation of cardiomyocyte integrity. These results unveil the BMP4–gremlin axis as a druggable pathway for the treatment of myocardial inflammation, limiting the severe sequelae of cardiac fibrosis and heart failure.

## Abstract (web summary)

Perez-Shibayama et al. report that BMP4 signaling is downregulated in autoimmune myocarditis. Restoring BMP4 levels with antibodies that neutralize the BMP inhibitors gremlin-1 and gremlin-2 can ameliorate cardiac inflammation and improve function in mouse models.

## Main

Myocarditis is a prototypic inflammatory heart disease and one of the most common causes of myocardial damage in patients with suspected myocardial infarction but non-obstructive coronary arteries^1,2^. Acute myocardial inflammation develops into chronic, potentially lethal inflammatory cardiomyopathy in 20–30% of patients^3,4^. Currently, treatments for acute and chronic myocardial inflammation have limited effectiveness and vary from restriction of physical activity, anti-phlogistic treatment for patients with pericardial involvement and general heart failure treatment to off-label use of immunosuppressive medication^4,5^. Despite the identification of a plethora of pathways leading to myocardial inflammation^6^, the elucidation of effective therapeutic intervention remains incomplete because the overarching molecular mechanisms that govern the balance between myocardial homeostasis and inflammation are incompletely understood.

One of the major causes underlying tissue damage during human myocarditis is the generation of autoimmune T cell responses against major histocompatibility complex (MHC) class II-binding peptides derived from the contractile protein myosin heavy chain 6 (MYH6)^7,8^. The spontaneous development of progressive myocarditis in transgenic mice expressing particular human MHC class II molecules^8,9^ supports the notion that MHC class II-dependent antigen presentation to CD4^+^ T cells is a key process that drives myocardial inflammation under various conditions^10,11^. Moreover, the development of myocarditis in patients with cancer treated with immune checkpoint inhibitors underscores the severe immunopathological damage caused by unleashing both CD4^+^ and CD8^+^ T cell reactivity against self-antigens in the cardiac microenvironment^12–15^.

T cell activation and differentiation, in both lymphoid organs and inflamed tissues, are controlled in distinct niches that are underpinned by specialized fibroblasts that interact with immune cells^16–18^. Because fibroblastic stromal cells constitute approximately 20% of non-cardiomyocytic cells in the healthy heart^19,20^, it is conceivable that changes in fibroblast activation states during inflammatory processes affect the functionality of T cells in the diseased cardiac microenvironment. Moreover, reprogramming of fibroblasts during their interaction with immune cells most likely determines the nature and extent of repair processes because these cells coordinate revascularization and remodeling of the tissue leading to preservation of the cardiac architecture and function^11,21^. Importantly, in patients with heart failure with or without reduced ejection fraction, the extent of fibrotic changes is a strong predictor for adverse outcome with higher mortality and increased hospitalization rate^22,23^. Hence, it is important to identify druggable molecular pathways that control the interplay between immune cells and cardiac fibroblasts.

Recent single-cell transcriptomics-based analyses of healthy and diseased human cardiac tissues revealed that inflammation is a common denominator associated with a wide range of cardiomyopathies and heart failure^24–26^. The interaction of immune cells with non-hematopoietic cells in the cardiac microenvironment is regulated by surface molecules and soluble factors that act at short range. For example, tissue cytokines belonging to the transforming growth factor-β (TGFβ) superfamily, including bone morphogenetic proteins (BMPs), are of particular importance for the homeostasis and functional preservation of the cardiac tissue^27^. Although BMP signaling appears to be involved in the remodeling process of the injured heart^28,29^, surprisingly little is known about the role of this pathway in cardiac inflammation and fibrosis. In this study, we used single-cell transcriptomics of inflamed human and murine heart tissue to assess whether and to what extent dysregulation of BMPs is associated with myocardial inflammation and cardiac tissue integrity. We found that the BMP4–Gremlin-1/2 (GREM1/2) axis is a key pathway involved in the maintenance of homeostatic heart function. Antibody-mediated neutralization of both GREM1 and GREM2 reinvigorated BMP signaling in the inflamed myocardium and, thereby, attenuated immune cell activity, reduced fibrotic remodeling and preserved cardiomyocyte integrity. In sum, selective restoration of BMP signaling in the inflamed heart facilitates treatment of myocardial inflammation and, thereby, efficiently diminishes cardiac fibrosis and prevents heart failure.

## Results

### Cellular interactions during T cell-driven myocarditis

**Fig. 1:**
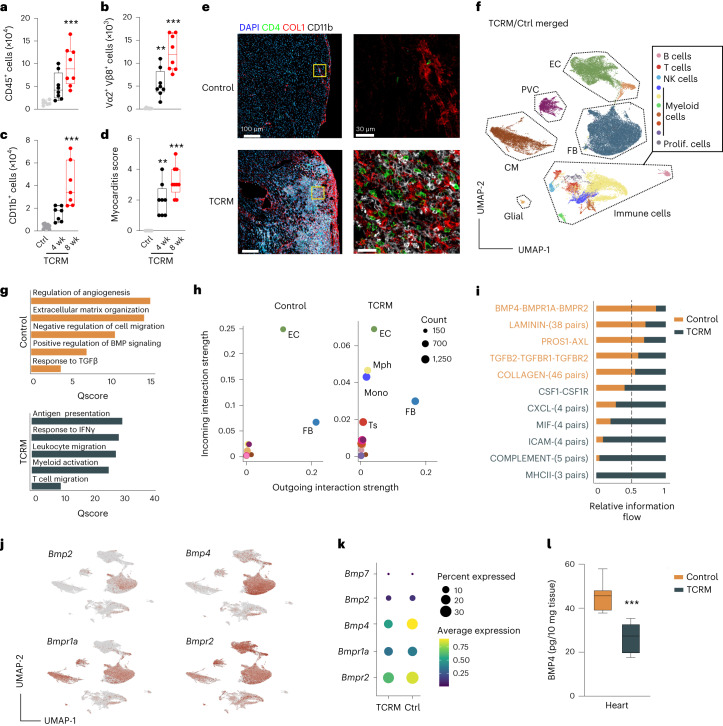
Cellular interactions in autoimmune myocarditis. **a**–**c**, Enumeration of heart-infiltrating CD45^+^ cells (**a**), MYH6-specific (Vα2^+^ Vβ8^+^CD4^+^) T cells (**b**) and CD11b^+^ myeloid cells (**c**) from 4-week-old and 8-week-old TCRM or control (Ctrl) mice. **d**, Histopathological disease severity in control and TCRM mice. **e**, Representative confocal microscopy images showing CD4^+^ and CD11b^+^ cells and COL1 deposition in hearts from 8-week-old control and TCRM mice. Boxed areas in left panels denote magnified area in right panels (*n* = 4). **f**–**k**, scRNA-seq and snRNA-seq analysis from total cardiac cells from age-matched and sex-matched control and TCRM mice. **f**, UMAP representation showing 16 cell populations in the heart of control and TCRM mice. **g**, Significantly enriched pathways according to GO enrichment analysis based on differentially expressed genes between total cardiac cells from control and TCRM mice. **h**, Scatter plot indicating incoming and outgoing interaction strengths of individual cell subsets based on network centrality measures on the aggregated cell–cell communication network. Dot size reflects interaction count. **i**, Top significantly enriched ligand–receptor pairs grouped according to functional similarity. Numbers in brackets indicate the quantity of receptor pairs within a functional group. **j**, UMAP projection showing the expression of the indicated genes. **k**, Dot plot depicting the average expression of the indicated BMP pathway-related genes in cardiac cells. **l**, Quantification of BMP4 protein in heart homogenates by ELISA. scRNA-seq/snRNA-seq data represent a total of 28,913 cells per nuclei from control (*n* = 6) and TCRM (*n* = 6) mice. Pooled data from 2–3 independent experiments with *n* = 7, 8 and 8 mice per group (**a**–**c**), *n* = 8, 8 and 9 mice per group (**d**) or *n* = 6 mice per group (**l**). Representative sections from one out of five control or TCRM mice (**c**). Dots represent values from individual mice; box and whiskers show minimum to maximum, mean ± interquartile range (**a**–**d** and **l**). CM, cardiomyocytes; EC, endothelial cells; FB, fibroblasts; Mono, monocytes; Mph, macrophages; NK cells, natural killer cells; PVC, perivascular cells; Ts, T cells; wk, weeks. Source data

**Extended Data Fig. 1:**
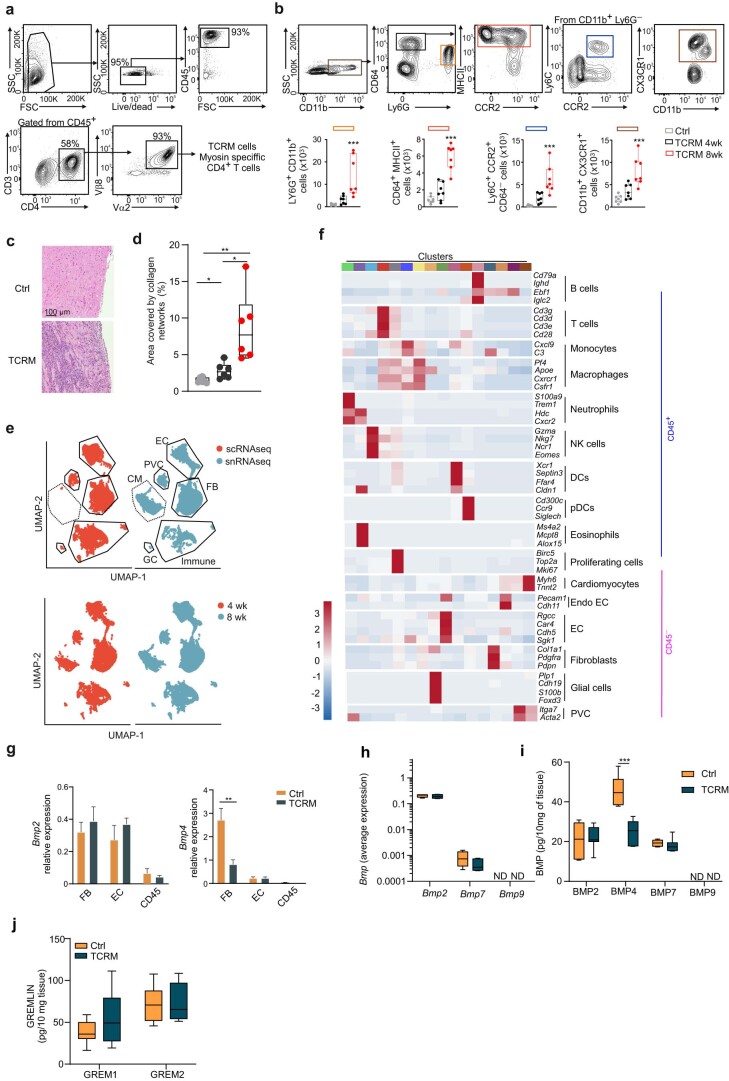
(**a**) Gating strategy for flow cytometric characterization of MYH6-specific cardiac T cells expressing the Vα2 and Vβ8 TCR chains. (**b**) Gating strategy for flow cytometric characterization of heart-infiltrating myeloid cells (upper panel) with colored frames indicating specific monocyte/macrophage populations analyzed in TCRM and transgene-negative littermate control mice at the age of 4 and 8 weeks (N = 7 mice per group from at 2 independent experiments). (**c**) Representative microscopy images from hematoxylin&eosin-stained sections from 8-week-old Ctrl and TCRM mice. (**d**) Quantification of picrosirius red-stained collagen networks on heart sections (N = 6 mice per group from 2 independent experiments). (**e**) UMAP representation of cardiac cell populations derived from sc- and snRNA-seq (upper panel) and of 4- and 8-week-old mice (bottom panel). (**f**) Heatmap showing the expression of marker genes used to assign cell identity to cardiac cells from Ctrl and TCRM mice detected by sn- and scRNA-seq analysis. (**g**) *Bmp2* and *Bmp4* mRNA expression as determined by RT-PCR in the indicated FACS-sorted cardiac cells from 8-week-old Ctrl or TCRM mice; FB, CD45^–^ PDPN^+^ CD31^–^ fibroblast; EC, CD45^–^ PDPN^–^ CD31^+^ endothelial cells; CD45, CD45^+^ immune cells. Bars show mean ± SEM (N = 6 mice per group from 3 independent experiments). (**h**) mRNA expression of the indicated BMPs from cardiac fibroblasts measured by sc/snRNAseq (N = 5 from 2 independent experiments). (**i**) Concentrations of the corresponding BMPs from cardiac homogenates measured by ELISA (N = 6 mice per group from two independent experiments). (**j**) GREM1 and GREM2 concentrations measured by ELISA from cardiac homogenates from 8-week-old littermate controls (Ctrl) or TCRM mice; N = 6, mice per group data from 2 independent experiments. Box and whiskers show min to max, mean ± interquartile range. Statistical analysis was performed using Student’s t test (g-j); one-way ANOVA with Dunnett’s multiple comparison test (b and d) with *, p < 0.05; **, p < 0.01; ***, p < 0.001. Source data

Activation of cardiac antigen-specific T cells and migration of effector T cells into the myocardium is a hallmark of inflammatory myocardial disease^7,10,14^. The full spectrum of T cell-mediated myocardial inflammation, ranging from acute myocarditis and chronic inflammatory cardiomyopathy to heart failure, can be recapitulated in T cell receptor transgenic mice with more than 95% of the CD4^+^ T cell pool recognizing the immunodominant MYH6_614–629_ epitope (TCRM mice)^7,30^. In this model, CD45^+^ immune cell accumulation in the heart muscle rapidly increases between 4 weeks and 8 weeks of age (Fig. 1a), with cardiac antigen-specific CD4^+^ T cells (Fig. 1b and Extended Data Fig. 1a) and inflammatory macrophages and other myeloid cells (Fig. 1c and Extended Data Fig. 1b) forming the main fractions of the infiltrate. Histopathological disease severity determined on hematoxilin and eosin (H&E)-stained heart sections (Extended Data Fig. 1c) increased with age (Fig. 1d), leading to large focal accumulations of CD4^+^ T cells and CD11b^+^ myeloid cells in 8-week-old TCRM mice in comparison to non-transgenic littermate control mice (Fig. 1e). Inflamed areas were underpinned by collagen-1 (COL1)-positive fibroblasts (Fig. 1e), which resulted in a significant increase of collagen networks in hearts of 8-week-old TCRM mice (Extended Data Fig. 1d). These data indicate that the cellular landscape during the early phase of CD4^+^ T cell-mediated myocarditis changes rapidly with immediate involvement of extracellular matrix (ECM)-producing cardiac fibroblasts.

To dissect the molecular circuits underlying the cellular changes during progressive myocardial inflammation, we performed droplet-based single-cell RNA sequencing (scRNA-seq) and single-nucleus RNA sequencing (snRNA-seq) analysis of cardiac cells from TCRM and transgene-negative littermate control mice at 4 weeks and 8 weeks of age (Extended Data Fig. 1e). Unsupervised clustering and uniform manifold and projection (UMAP) visualization in combination with cluster identification using established marker genes^24–26^ (Extended Data Fig. 1f and Supplementary Table 1) revealed the presence of different immune cell populations and non-hematopoietic cells (Fig. 1f). Cardiomyocytes, identified by the expression of *Myh6* and *Tnnt2*, could be detected only by snRNA-seq (Extended Data Fig. 1e, top) because the large cell body precludes droplet encapsulation. All other cardiac cell populations were equally well represented in scRNA-seq and snRNA-seq analyses in TRCM and control mice (Extended Data Fig. 1e, bottom). Single-cell transcriptomics data of cardiac cells from 4-week-old and 8-week-old TCRM mice and age-matched and sex-matched healthy control mice were analyzed to infer the strength and patterns of intercellular communication. Pathway enrichment analyses based on differentially expressed genes denoted high activity of molecular circuits regulating angiogenesis, ECM organization and cell migration in healthy control hearts (Fig. 1g, top). In addition, the data indicated that BMP pathways and responses to TGFβ support homeostatic cellular functions in the heart (Fig. 1g, top). Inflammation profoundly changed the transcriptional state of cardiac cells with increased activity of immunological pathways, such as antigen presentation, response to interferon-γ (IFNγ), immune cell migration and myeloid cell activation (Fig. 1g, bottom). Cell–cell communication network assessment using the CellChat toolkit^31^ highlighted cardiac fibroblasts as the strongest source of intercellular signals in both healthy and inflamed hearts, whereas endothelial cells appeared as the main signal receivers (Fig. 1h). Furthermore, the analysis indicated that T cells, monocytes and macrophages increased their participation in cellular interactions during inflammation, mainly as signal receivers (Fig. 1h, right). Further cell–cell communication analysis of fibroblasts interacting with neighboring cells predicted that MHC class II-dependent cellular interactions of cardiac fibroblasts occur almost exclusively in inflamed hearts, whereas the binding of *Bmp4* to the heterotetrameric BMP receptor 1a and 2 (*Bmpr1a*–*Bmpr2*) as ligand–receptor interaction showed the strongest relative decrease during myocarditis compared to homeostatic hearts (Fig. 1i). Indeed, cardiac fibroblasts appeared as the main source of *Bmp4* mRNA, whereas the receptor showed a broader distribution (Fig. 1j). Fibroblasts exhibited low *Bmp2* and *Bmp7* mRNA expression, whereas *Bmp9* mRNA expression was undetectable (Fig. 1k and Extended Data Fig. 1h). T cell-driven myocardial damage resulted in a significant reduction of BMP4 protein concentration in heart homogenates of TCRM mice in comparison to control hearts (Fig. 1l), whereas BMP2 and BMP7 protein concentrations were not significanltly changed (Extended Data Fig. 1i). Likewise, the concentrations of GREM1 and GREM2 protein were not affected by the inflammation in TCRM hearts in comparison to healthy control hearts (Extended Data Fig. 1j). The decrease of both *Bmp4* mRNA expression (Fig. 1k) and BMP4 protein (Fig. 1l), together with the fibroblast-restricted regulation of *Bmp4* during myocardial inflammation, further suggest that BMP4 produced by fibroblasts serves as a key rheostat of cellular interactions in the cardiac microenvironment.

### Restriction of BMP4 availability in the inflamed myocardium

**Fig. 2:**
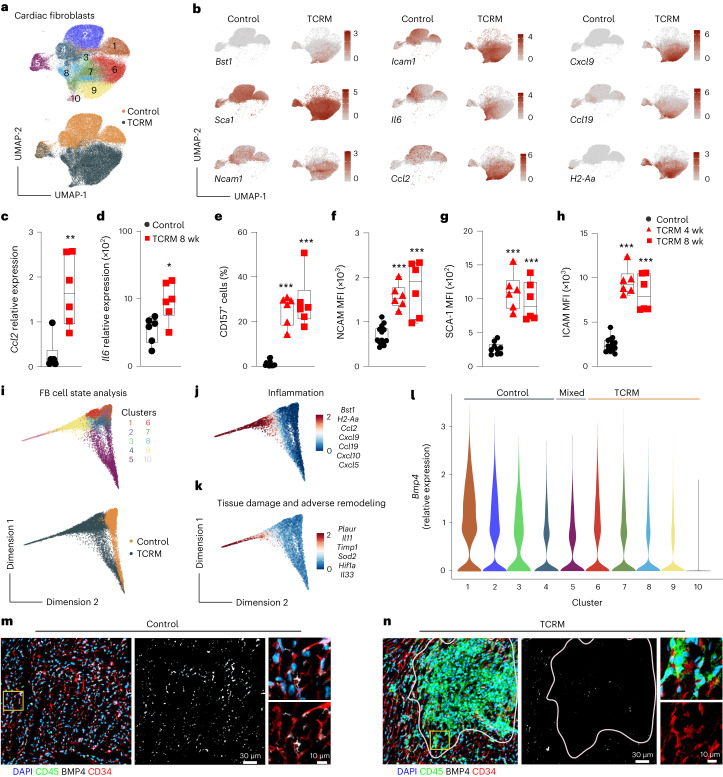
Cardiac fibroblast-mediated regulation of BMP4 availability in the inflamed myocardium. **a**,**b**,**i**–**l**, sc-RNA-seq analysis of sorted CD45^−^PDPN^+^CD31^−^ cardiac fibroblasts isolated from 4-week-old and 8-week-old control or TCRM mice. **a**, UMAP representation showing 10 cell clusters depicting cardiac fibroblast heterogeneity (top) and condition (bottom). **b**, Projection of the indicated gene expression on cardiac fibroblast UMAP plot from control and TCRM mice. **c**,**d**, *Ccl2* (**c**) and *Il6* (**d**) mRNA expression in sorted cardiac fibroblasts of 8-week-old control or TCRM mice. **e**–**h**, Multi-parametric flow cytometric analysis of surface expression of CD157 (**e**), NCAM (**f**), SCA-1 (**g**) and ICAM1 (**h**) by cardiac fibroblasts from control or TCRM mice. MFI of the indicated activation markers in CD45^−^PDPN^+^CD31^−^ cardiac fibroblasts. Dots represent values from individual mice;box plots as in Fig. 1. **i**, Diffusion map representation based on gene expression in cardiac fibroblasts colored according to heterogeneity (top) and origin (bottom). **j**,**k**, Projection of the indicated gene signatures onto diffusion maps. **l**, Expression profile of *Bmp4* in cardiac fibroblast subsets shown as violin plots. **m**,**n**, Representative confocal microscopy analysis of hearts from control (**m**) and TCRM (**n**) mice using the indicated markers. Single-cell transcriptomics data represent a total of 65,545 cardiac fibroblasts from control (*n* = 4) and TCRM (*n* = 5) mice. Pooled data from 2–3 independent experiments with *n* = 6 mice per group (**c**) or *n* = 9, 6 and 6 mice per group (**d**–**h**). Representative sections from one out of five control or TCRM mice (**m**,**n**). Statistical analysis was performed using two-tailed Student’s *t*-test (**c**,**d**) or one-way ANOVA with Tukey’s multiple comparisons test (**e**–**h**) with *P < 0.05, **P < 0.01 and ***P < 0.001. Source data

**Extended Data Fig. 2:**
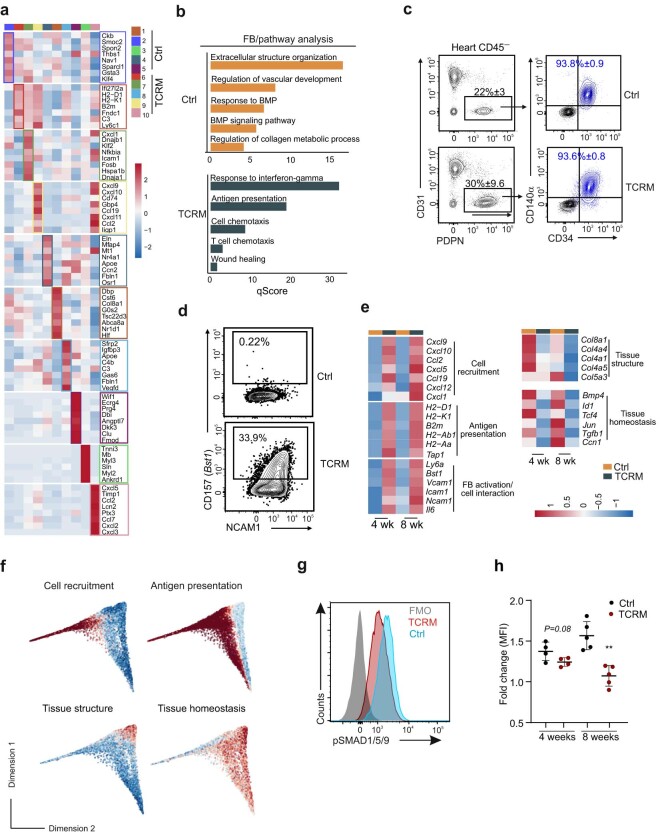
Single cell RNAseq analysis and flow cytometric characterization of CD45^–^ PDPN^+^ CD31^–^ cardiac fibroblasts isolated from 4- and 8-week-old Ctrl and TCRM mice. (**a**) Heatmap showing the expression of the top differentially expressed genes from the 10 clusters of cardiac fibroblasts from Ctrl and TCRM mice. (**b**) Significantly enriched pathways according to Gene Ontology (GO) enrichment analysis based on differentially expressed genes between Ctrl and TCRM cardiac fibroblasts. (**c**) Flow cytometric gating strategy used to characterize cardiac fibroblast phenotype. (**d**) Representative density plot depicting the expression of CD157 and NCAM by cardiac fibroblasts from Ctrl and TCRM mice. (**e**) Differentially regulated functional pathways from single cell RNAseq analysis of cardiac fibroblasts. (**f**) Expression pattern of genes assigned to the indicated cellular processes projected onto diffusion maps per signature as in panel (e). (**g**, **h**) Intracellular phospho-SMAD1/5/9 expression by CD45^−^ CD31^−^ PDPN^+^ CD56^+^ cardiac fibroblasts isolated from 4- (N = 4 mice per group) and 8-week-old (N = 5 mice per group) Ctrl and TCRM mice. (g) Representative histogram from cells isolated from 8-week-old mice. (h) Fold change of pSMAD1/5/9 mean fluorescence intensity (MFI) was calculated relative to baseline pSMAD1/5/9 expression in CD56^−^ fibroblasts; pooled data from two independent experiments. Dots indicate individual mice, mean ± SEM is displayed. Statistical analysis was performed using Two-tailed Student’s t test (h). with **, p < 0.01. Source data

Because BMP4 availability during myocardial inflammation appears to be controlled through reprogramming of fibroblast activity, we sorted cardiac fibroblasts from hearts of 4- and 8-week-old TCRM and control mice and performed scRNA-seq analysis. Unsupervised clustering and UMAP visualization revealed 10 different clusters (Fig. 2a, top) that exhibited distinct transcriptional profiles (Extended Data Fig. 2a). Only cluster 5 was shared between cardiac fibroblasts from TCRM and healthy control mice (Fig. 2a, bottom), indicating that myocardial inflammation leads to a profound change in the activation state of fibroblasts. Pathway analysis of single-cell transcriptomics data from cardiac fibroblasts confirmed that BMP signaling is a key trait of homeostatic cellular interactions that is overwritten by immune pathways during myocardial inflammation (Extended Data Fig. 2b). Projection of selected gene activities that signify fibroblast–immune cell interaction^16,32–34^ showed markedly elevated expression in fibroblasts from TCRM hearts (Fig. 2b). Quantitative RT–PCR analysis of sorted cardiac fibroblasts confirmed the increased expression of the inflammatory monocyte-attracting chemokine *Ccl2* (Fig. 2c) and the inflammatory cytokine *Il6* (Fig. 2d) during autoimmune T cell-driven myocarditis. The change of a large fraction of cardiac fibroblasts from a homeostatic to an inflammatory phenotype of cardiac fibroblasts was further validated by multi-parametric flow cytometry (Extended Data Fig. 2c) in TCRM and control mice of 4 weeks and 8 weeks of age. We found a significant increase of CD157 (encoded by *Bst1*)-expressing fibroblasts (Fig. 2e and Extended Data Fig. 2d) and significant upregulation of the fibroblast activation molecules NCAM1 (Fig. 2f), SCA-1 (Fig. 2g) and ICAM1 (Fig. 2h) early after onset of myocarditis, supporting the notion that fibroblasts can change swiftly from a homeostatic to an inflammatory phenotype during myocardial inflammation.

To identify fibroblast subsets and major molecular pathways associated with inflammation-driven fibroblast activation, we used diffusion maps as a nonlinear dimensionality reduction technique to infer the differentiation of cellular processes according to changes in transcriptional states. We found that cells from the common cluster 5 differentiate either toward the homeostatic fibroblast clusters 1, 2 and 4 or toward the fibroblast subsets 8–10 present in inflamed TCRM hearts (Fig. 2i). Accordingly, the projection of well-known inflammatory fibroblast markers (Fig. 2b) highlighted clusters 9 and 10 as main inflammatory fibroblast populations (Fig. 2j). Computation of differentially expressed genes in fibroblasts from TCRM and control hearts (Extended Data Fig. 2e) and extended functional pathway analysis revealed rapid upregulation of genes involved in immune cell recruitment, antigen presentation and fibroblast–immune cell interaction (Extended Data Fig. 2f). In contrast, molecular pathways mediating tissue integrity and homeostasis were downregulated in cardiac fibroblasts of 4-week-old TCRM mice (Extended Data Fig. 2f). Projection of these pathways onto diffusion maps indicated that (1) fibroblasts in cluster 1 are particularly engaged in tissue integrity and homeostasis; (2) fibroblasts in clusters 5–10 upregulate their antigen presentation machinery; and (3) increased immune cell recruitment is mainly a trait of the inflammatory fibroblasts in clusters 9 and 10 (Extended Data Fig. 2f). Moreover, differentially regulated genes that signify tissue damage and adverse fibroblast remodeling were as well mainly expressed in inflammatory fibroblast subsets (Fig. 2k). Consistent with the loss of *Bmp4* expression under inflammatory conditions (Fig. 1k,l), we found that fibroblasts in clusters 9 and 10 showed the strongest *Bmp4* downregulation (Fig. 2l). To assess to what extent reduced BMP4 concentration in the inflamed myocardium affects signaling events downstream of the BMPR1A/BMPR2 complex in cardiac fibroblasts, we determined changes in SMAD1/5/9 phosphorylation. We found significantly reduced SMAD1/5/9 phosphorylation in cardiac fibroblasts from 8-week-old TCRM mice in comparison to non-transgenic littermate control mice (Extended Data Fig. 2g,h), further supporting the notion that BMP4-mediated signaling in cardiac fibroblasts is a key pathway that controls fibroblast activity in the cardiac microenvironment. High-resolution confocal microscopy confirmed that BMP4 protein was expressed widely in cardiac fibroblasts of healthy control hearts (Fig. 2m). During myocardial inflammation, we found a substantial loss of BMP4 signal in fibroblasts underpinning immune cell aggregates (Fig. 2n), supporting the conclusion that accumulation of auto-aggressive immune cells in the myocardium and concomitant fibrotic remodeling processes lead to the local restriction of BMP4 availability and function in the myocardium.

### BMP4 regulates cardiac fibroblast activity

**Fig. 3:**
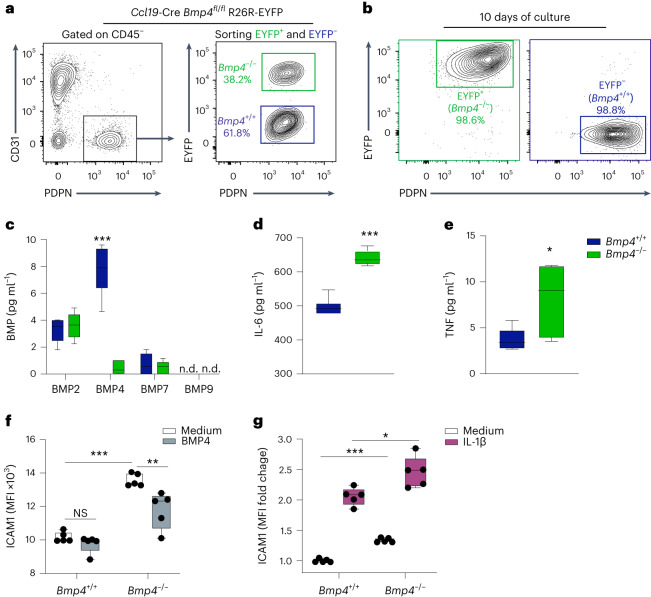
BMP4 regulation of cardiac fibroblasts. Cardiac fibroblasts were isolated from hearts of 8-week-old *Ccl19*-Cre R26R-EYFP mice. **a**, Representative dot plots showing the gating strategy for FACS of PDPN^+^CD31^−^, *Bmp4*-deficient (EYFP^+^) and *Bmp4*-proficient (EYFP^−^) cardic fibroblasts. **b**, Purity of *Bmp4*-deficient (EYFP^+^) and *Bmp4*-proficient (EYFP^−^) cardiac fibroblasts after 10 d of culture. Representative dot plots from three independent isolations. **c**, Production of the indicated BMPs by *Bmp4*^−/−^ (EYFP^+^) or *Bmp4*^*+/+*^ (EYFP^−^) fibroblasts. **d**,**e**, Production of the cytokines IL-6 (**d**) and TNF (**e**) by *Bmp4*^−/−^ (EYFP^+^) or *Bmp4*^*+/+*^ (EYFP^−^) fibroblasts after 24 h of culture (*n* = 5; pooled data from independent wells from two independent experiments). **f**,**g**, Expression of ICAM1 by *Bmp4*^−/−^ (EYFP^+^) or *Bmp4*^*+/+*^ (EYFP^−^) fibroblasts after exposure to medium or BMP4 (10 ng ml^−1^) (**f**) or IL-1β (1 ng ml^−1^) (**g**) as assessed by flow cytometry with MFI of ICAM1 in **f** and MFI fold change relative to ICAM1 expression by untreated *Bmp4*^*+/+*^ fibroblasts in **g**. Dots represent values from individual wells; pooled data of two independent experiments (*n* = 5). All box plots as in Fig. 1. Statistical analysis was performed using Student’s *t*-test (**c**–**e**) or one-way ANOVA with Dunnett’s multiple comparison test (**f**,**g**) with *P < 0.05, **P < 0.01 and ***P < 0.001. FACS, fluorescence-activated cell sorting. Source data

**Extended Data Fig. 3:**
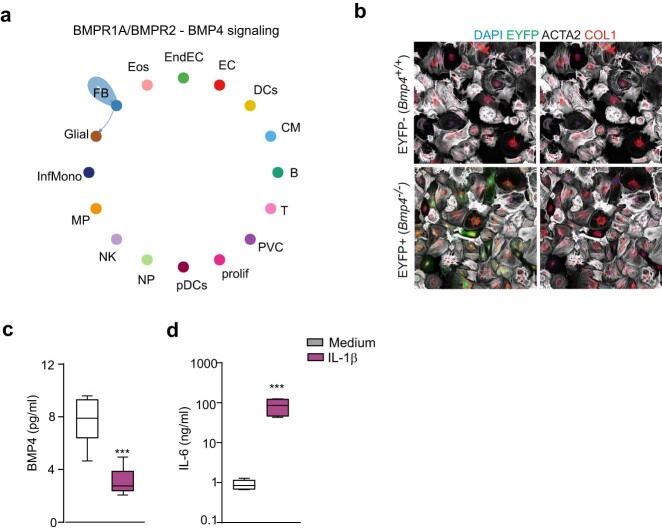
Activation of cardiac fibroblasts. (**a**) Computationally predicted murine BMP4-BMPR1a/BMPR2 interactions by cells in the homeostatic and inflamed cardiac microenvironment based on sc/snRNA-seq data using the CellChat tool. (**b**) Representative confocal images of *Bmp4*-deficient (EYFP^+^) and *Bmp4*-proficient (EYFP^−^) fibroblasts after 10 days of culture. (**c**, **d**) Production of BMP4 (c) and IL-6 (d) measured by ELISA from *Bmp4*-proficient (EYFP^−^) fibroblasts cardiac fibroblasts exposed IL-1β (1 ng/ml) or left untreated (medium) for 24 h. Box and whiskers show min to max, mean ± interquartile range. (N = 6; pooled data from two independent experiments). Statistical analysis was performed using Student’s t test (c-d) with ***, p < 0.001. Source data

To determine how changes in BMP4 concentration in the inflamed myocardium affect fibroblast function, we used bioinformatics analysis as indicator for BMP-mediated intercellular communication. Based on our scRNA-seq and snRNA-seq data from all cells in the cardiac microenvironment, the assessment of possible ligand–receptor interactions using the CellChat tool predicted that BMP4 is an autocrine regulator of fibroblast activity (Extended Data Fig. 3a). To further elaborate the regulatory circuits underlying autocrine BMP4-dependent control of fibroblast activity, we sorted *Ccl19*-Cre lineage-traced cardiac fibroblasts taking advantage of the fact that approximately 40% of PDPN^+^ cardiac fibroblasts express the Cre recombinase as shown by the expression of the enhanced yellow fluorescent protein (EYFP) when the R26R-EYFP reporter strain has been crossed with *Ccl19*-Cre mice (Fig. 3a). In addition, we crossed *Ccl19*-Cre R26R-EYFP mice with *Bmp4* floxed mice, facilitating the separation of EYFP-positive, *Bmp4*-deficient and EYFP-negative, *Bmp4*-proficient cardiac fibroblasts (Fig. 3a) that can be cultured in vitro for further analysis (Fig. 3b and Extended Data Fig. 3b). As expected, *Bmp4*-proficient cardiac fibroblasts exposed to inflammatory stimuli, such as IL-1β, downregulated BMP4 protein production (Extended Data Fig. 3c) and increased secretion of inflammatory mediators, such as IL-6 (Extended Data Fig. 3d). Production of BMP2, BMP7 or BMP9 by cardiac fibroblasts was not affected by the selective *Bmp4* deficiency (Fig. 3c), whereas IL-6 (Fig. 3d) and TNF (Fig. 3e) production was significantly elevated in *Bmp4*-deficient fibroblasts when compared to *Bmp4*-proficient fibroblasts. Likewise *Bmp4*-deficient fibroblasts showed elevated expression of the adhesion molecule ICAM1 in comparison to *Bmp4*-proficient fibroblasts (Fig. 3f,g). Exposure of cardiac *Bmp4*-proficient and *Bmp4*-deficient fibroblasts to IL-1β increased ICAM1 expression (Fig. 3g), whereas incubation with BMP4 reduced ICAM1 expression mainly in *Bmp4*-deficient fibroblasts (Fig. 3f). These data further support the notion that BMP4 is an autocrine tissue factor that maintains the homeostatic functions of cardiac fibroblasts.

### Restoration of BMP4-mediated signaling ameliorates myocarditis

**Extended Data Fig. 4:**
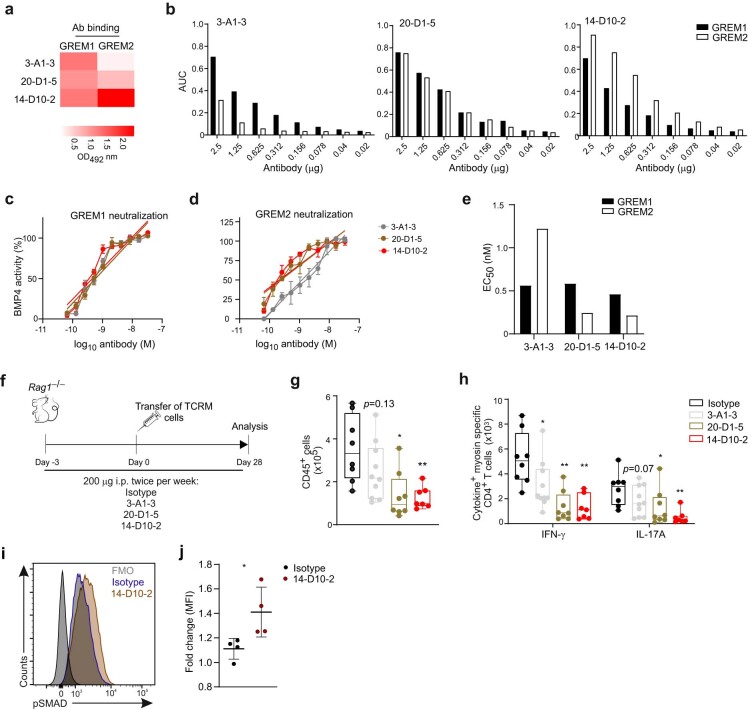
Binding and functional characterization of anti-GREM1/2 monoclonal antibodies. (**a**) Heatmap representing binding of 3-A1-3, 20-D1-5 and 14-D10-2 mAbs to human GREM1 and GREM2 as determined by ELISA. (**b**) Dose-dependent binding of the indicated anti-GREM1/2 mAbs to human GREM1 and GREM2 as determined by ELISA. Area under the curve (AUC) was calculated as the area under the titration curve from 1:2 serial dilutions of each antibody (2.5 - 0.02 µg) and GREM1 and GREM2 (0.5–0.008 µg/ml). (**c–e**) Neutralization capacity of the indicated mAbs using GREM1 and GREM2 in BMP4-induced luciferase activity by SL-0051 cells. Hundred percent BMP4 activity was determined as relative light units in the absence of GREM1 (c) or GREM2 (d) (N = 3, mean ± SEM; representative data from 1 out of 3 independent experiments with similar results). (e) The concentration of the indicated mAb necessary to restore 50% of BMP4 activity (EC_50_) was determined based on the values shown in (c) and (d); N = 6 independent replicates from 2 independent experiments. (**f–h**) Effect of anti-GREM1/2 mAb treatment in the adoptive T cell transfer myocarditis model. (f) Schematic representation of the experimental set up. (g) Quantification of heart-infiltrating CD45^+^ cells (h) and cytokine production of heart-infiltrating MYH6-specific CD4^+^ Vβ8^+^ T cells in *Rag1*^−/−^ mice at day 28 post T cell adoptive transfer. N = 8, 10, 8 or 7 mice per group from 3 independent experiments. Box and whiskers show min to max, mean ± interquartile range; dots indicate individual mice. (**i**, **j**) Intracellular phospho-SMAD1/5/9 expression by CD45^−^ CD31^−^ PDPN^+^ CD56^+^ cardiac fibroblasts isolated from 8-week-old TCRM mice treated with IgG2b isotype control or GREM1/2-neutralizing antibody (14-D10-2) with representative histograms shown in (i). (j) Fold change of pSMAD1/5/9 mean fluorescence intensity (MFI) calculated relative to baseline pSMAD1/5/9 expression in CD56^−^ fibroblasts (N = 4 mice per group from 2 independent experiments) Dots indicate individual mice, mean ± SEM is displayed. Statistical analysis was performed using one-way ANOVA with Dunnett’s multiple comparison test (g-h) or two tailed Student’s t test (j) with *, p < 0.05; **, p < 0.01; ***, p < 0.001. Source data

To determine the functional relevance of reduced BMP4 availability in the inflamed myocardium, we generated monoclonal antibodies that block the BMP inhibitors GREM1 and GREM2 and can, thereby, increase local BMP4 concentration. Out of the 42 antibodies generated by immunizing mice with human GREM1, we selected 3 IgG-secreting clones with different binding patterns to GREM1 and GREM2 (Extended Data Fig. 4a,b) for further in-depth characterization. The three antibodies neutralized GREM1 and GREM2 in vitro, with clone 14-D10-2 showing the highest efficacy (that is, the lowest half-maximal effective concentration (EC_50_) value) in restoring BMP4 activity (Extended Data Fig. 4c–e). We next used adoptive transfer of TCRM splenocytes into *Rag1*-deficient mice^7^ (Extended Data Fig. 4f) to determine the in vivo efficacy of the three selected monoclonal anti-GREM1/2 antibodies. We found that neutralization of both GREM1 and GREM2 led to a significant reduction of myocardial inflammation (Extended Data Fig. 4g). The finding that clone 14-D10-2 most efficiently antagonized the production of the cardiopathogenic cytokine IL-17A by MYH6-specific CD4^+^ T cells (Extended Data Fig. 4h) indicated that pan-GREM inhibition is an important feature of monoclonal antibodies to pharmacologically restore BMP4 availability in the inflamed myocardium.

**Fig. 4:**
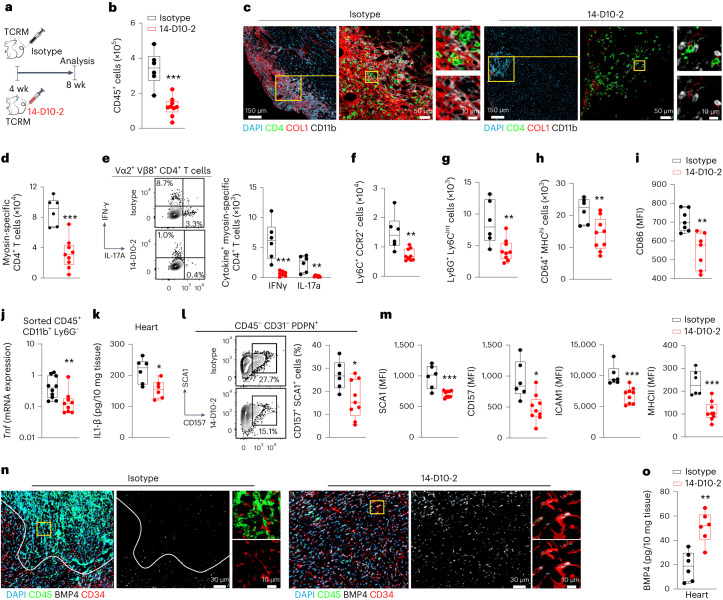
Therapeutic treatment of acute myocarditis in TCRM mice with anti-GREM 1/2 monoclonal antibody (14-D10-2). **a**, Schematic representation of the experimental design. **b**, Enumeration of CD45^+^ heart-infiltrating cells in 8-week-old TCRM mice treated with IgG2b isotype control or GREM1/2-neutralizing antibody (14-D10-2) using flow cytometry. **c**, Representative confocal microscopy images showing collagen deposition and immune infiltrates in the hearts of TCRM mice treated with isotype or 14-D10-2 antibodies. **d**,**e**, Flow cytometry-based enumeration (**d**) and functional characterization (**e**) of MYH6-specific, Vα2^+^Vβ8^+^CD4^+^ T cells with representative dot plots (left) and quantification of cytokine-producing MYH6-specific T cells (right). **f**–**h**, Flow cytometric enumeration of CD11b^+^Ly6C^+^CCR2^+^ inflammatory monocytes (**f**), CD11b^+^Ly6C^int^Ly6G^+^ neutrophils (**g**) and CD11b^+^CD64^+^MHCII^hi^ activated macrophages (**h**). **i**, MFI of CD86 expression on CD11b^+^CD64^+^MHCII^hi^ activated macrophages. **j**, *Tnf* mRNA expression by sorted CD11b^+^Ly6G^−^ myeloid cells. **k**, IL-1β protein concentration in cardiac homogenates. **l**, Fraction of CD157^+^SCA1^+^ cells of CD45^−^PDPN^+^CD31^−^ cardiac fibroblasts. **m**, MFI of the indicated activation markers by CD45^−^PDPN^+^CD31^−^ cardiac fibroblasts. **n**, Representative confocal microscopy images showing BMP4 production by CD34^+^ fibroblasts in inflamed hearts of isotype antibody-treated TCRM mice and restoration of BMP4 production by 14-D10-2 antibody treatment. **o**, Quantification of BMP4 protein in heart homogenates by ELISA. Box plots as in Fig.1; pooled data from three independent experiments with *n* = 6 (control) or *n* = 9 (TCRM) mice per group (**b**,**d**–**m**,**o**). Representative sections from one out of five control or TCRM mice (**c**,**n**). Statistical analysis was performed using Student’s *t*-test with *P < 0.05, **P < 0.01 and ***P < 0.001. Source data

In the next set of experiments, we treated 4-week-old TCRM mice with the pan-GREM inhibitory antibody 14-D10-2 for 4 weeks (Fig. 4a). Mice treated with anti-GREM1/2 antibody showed reduced accumulation of immune cells in the myocardium (Fig. 4b) with only small areas being underpinned by CD4^+^ T cells, CD11b^+^ myeloid cells and activated COL1^+^ fibroblasts (Fig. 4c). Flow cytometric analysis confirmed the significant reduction of MYH6-specific T cell accumulation in the myocardium of anti-GREM1/2-treated TCRM mice (Fig. 4d) and revealed the almost complete functional attenuation of cardiotropic CD4^+^ T cells with significantly reduced IFNγ and IL-17A production (Fig. 4e). Likewise, the accumulation of CD11b^+^Ly6C^+^CCR2^+^ inflammatory monocytes, CD11b^+^Ly6G^+^Ly6C^int^ neutrophils and CD11b^+^CD64^+^MHCII^hi^ macrophages was substantially reduced in 14-D10-2-treated TCRM mice (Fig. 4f–h). Decreased expression of CD86 by MHCII^high^ macrophages (Fig. 4i), diminished expression of *Tnf* mRNA by CD11b^+^Ly6G^−^ myeloid cells (Fig. 4j) and reduced IL-1β concentration in the heart (Fig. 4k) underscored the substantial attenuation of myeloid cell activity after 14-D10-2 treatment. Moreover, GREM1/2 blockade resulted in the reduction of inflammatory fibroblast activity with significantly lowered expression of CD157, SCA1, ICAM1 and MHCII (Fig. 4l,m). Treatment with anti-GREM1/2 antibody led to the resolution of inflammatory clusters in the myocardium of 8-week-old TCRM mice with continued provision of BMP4 by cardiac fibroblasts (Fig. 4n). The preservation of physiological BMP4 concentrations in TCRM hearts after 14-D10-2 treatment (Fig. 4o) and the elevated SMAD1/5/9 phosphorylation in cardiac fibroblasts (Extended Data Fig. 4i,j) underscore that antibody-mediated restoration of BMP4 availability and BMPR signaling in the cardiac microenvironment efficiently counteracts immune cell and fibroblast activation in the course of acute myocardial inflammation.

### GREM1/2 blockade prevents lethal inflammatory cardiomyopathy

**Fig. 5:**
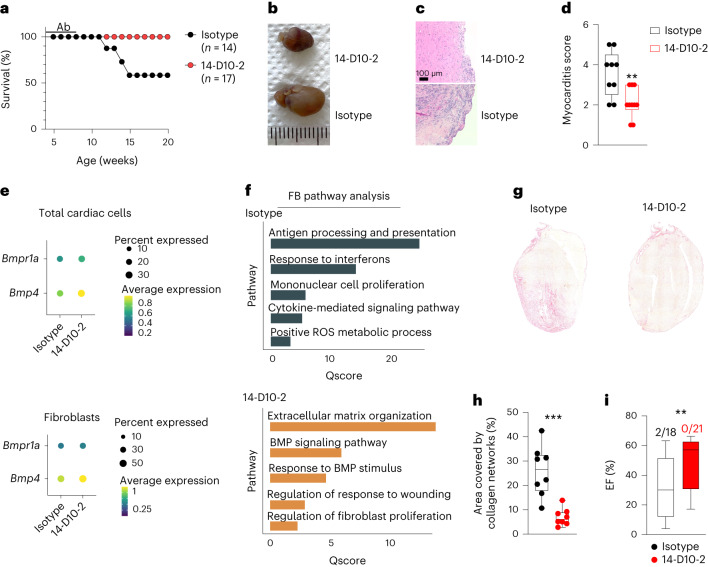
Long-term effects of GREM1/2 blockade. **a**, Survival of TCRM mice after treatment with GREM1/2-neutralizing antibody (14-D10-2) or IgG2b isotype control antibody. **b**–**d**, Cardiac gross pathology (**b**), representative H&E-stained heart sections (**c**) and histopathological disease severity (**d**) of 20-week-old TCRM mice subjected to the indicated treatment (*n* = 9 and 10 mice per group). **e**, Dot plots depicting the average expression of the indicated genes in total cardiac cells (top) and cardiac fibroblasts (FB; bottom) of 12-week-old TCRM mice treated with the indicated antibodies. **f**, Top significantly enriched pathways according to GO enrichment analysis based on differentially expressed genes in all cardiac cells from TCRM mice treated with control or GREM1/2-neutralizing antibodies. **g**,**h**, Representative images of picrosirius red-stained heart sections (representative image from eight mice per group) (**g**) and quantification of collagen network size (**h**) from 12-week-old TCRM mice treated with control or GREM1/2-neutralizing antibodies (*n* = 8 mice per group from three independent experiments). **i**, Ejection fraction (EF) as determined by echocardiography in 8-week-old TCRM mice treated with 14-D10-2 or isotype antibody between week 4 and week 8. Numbers indicate fraction of mice developing heart failure before the end of the treatment period; box plots as in Fig. 1 (*n* = 18 and 21 mice per group). scRNA-seq and snRNA-seq data represent a total of 27,160 cells per nuclei from control (*n* = 4) and TCRM (*n* = 4) mice (**e**,**f**). Statistical analysis was performed using Mann–Whitney *U*-test (**d**,**h**,**i**) with **P < 0.01 and ***P < 0.001. ROS, reactive oxygen species. Source data

To assess whether the short-term anti-GREM1/2 treatment of TCRM mice yields protection from chronic T cell-mediated myocardial damage, we monitored the mice for 20 weeks. Approximately 50% of the mice treated with the control antibody at the age of 4–8 weeks had to be euthanized between 12 weeks and 15 weeks of age due to severe clinical symptoms (dyspnea, severe lethargy or wasting) (Fig. 5a) and showed dilated hearts as determined by gross pathological examination (Fig. 5b). By contrast, all mice treated with anti-GREM1/2 survived (Fig. 5a) and did not show signs of dilated cardiomyopathy (Fig. 5b). H&E staining-based histopathological assessment of disease severity (Fig. 5c) at the age of 20 weeks revealed significantly reduced myocarditis scores in mice treated with 14-D10-2 compared to isotype treatment (Fig. 5d). These data indicate that short-term treatment with monoclonal anti-GREM1/2 antibodies at the onset of myocardial inflammation in TCRM mice provided protection against lethal inflammatory cardiomyopathy despite the persistence of high numbers of potentially cardiopathogenic CD4^+^ T cells.

**Extended Data Fig. 5:**
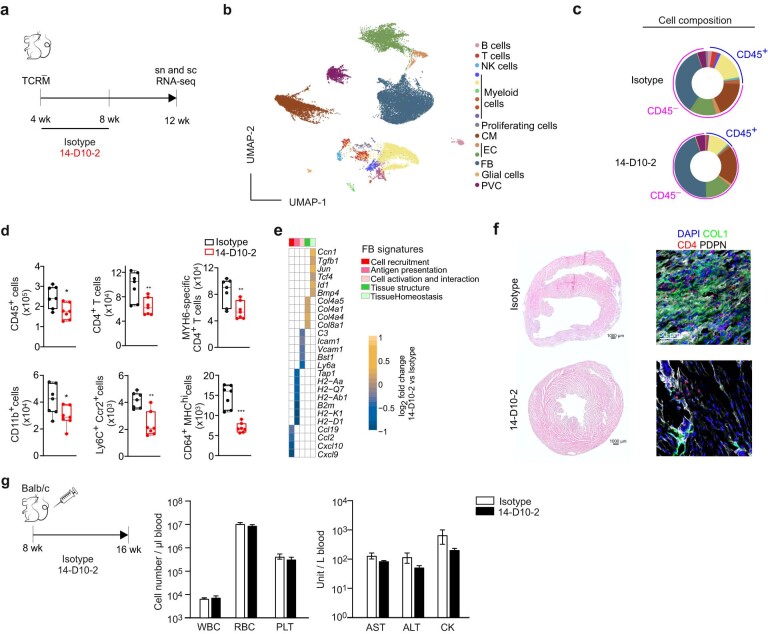
Single cell- and single nucleus RNA-seq analysis of cardiac cells from TCRM mice treated 4 for weeks with isotype control or GREM1/2-neutralizing antibody 14-D10-2 and further validation experiments. (**a**) Schematic representation of the experimental set up. (**b**) UMAP representation showing marker gene assigned cell populations in the murine heart. (**c**) Pie charts depicting the proportions of the different cell populations based on snRNA-seq analysis of TCRM hearts; isotype, 13,498 nuclei; 14-D10-2, 13,662 nuclei. (**d**) Enumeration of the indicated heart-infiltrating immune cell populations in 12-week-old TCRM mice treated with Ig2b isotype control or GREM1/2-neutralizing antibody (14-D10-2) using flow cytometry (N = 7 mice per group, pooled data from 2 independent experiments). Box and whiskers show min to max, mean ± interquartile range; dots indicate individual mice. (**e**) Heatmap showing changes in the expression of differentially expressed genes grouped by function from the fibroblast population in TCRM mice treated with isotype or 14-D10-2 antibodies. (**f**) Representative images showing immune cell infiltration and collagen deposition in hearts from 12-week-old TCRM mice treated with the indicated antibodies; hematoxylin-eosin (left panels) and confocal microscopy with indicated antibody staining (right panels). (**g**) Assessment of blood cell composition and the concentration of key serum enzymes in 16-week-old Balb/c mice according to the indicated treatment scheme; WBC, white blood cells; RBC, red blood cells; PLT, platelets; AST, aspartate aminotransferase; ALT, alanine transaminase; CK, creatine kinase (N = 6 mice per group, pooled data from two independent experiments; Bars indicate mean ± SEM). Statistical analysis was performed using Student’s t test (d, g, h) and 2-way Anova with Sidak’s multiple comparisons test (disease activity score in h), with *, p < 0.05; **, p < 0.01; ***, p < 0.001. Source data

To resolve the molecular processes that prevented progression of myocardial inflammatory disease after GREM1/2 blockade, we performed single-cell and single-nucleus transcriptome analyses of cardiac cells from 12-week-old TCRM mice—that is, before the onset of severe clinical symptoms (Extended Data Fig. 5a,b). Cell composition analysis based on the scRNA-seq and snRNA-seq data and additional flow cytometric analyses showed a significant reduction of the CD45^+^ immune cell infiltrate, including MHY6-specific CD4^+^ T cells and different myeloid cell subsets (Extended Data Fig. 5c,d). *Bmp4* mRNA expression was elevated in cardiac cells (Fig. 5e, top) and cardiac fibroblasts (Fig. 5e, bottom) of mice treated with the 14-D10-2 antibody. Gene set enrichment analysis confirmed that cardiac fibroblasts from isotype-treated mice remained in an inflamed state with significantly upregulated pathways, such as antigen presentation and response to interferons, whereas cardiac fibroblasts of TCRM mice treated with anti-GREM1/2 had regained homeostatic functions, including BMP signaling (Fig. 5f). Indeed, cardiac fibroblasts from TCRM mice treated with 14-D10-2 had recovered expression of molecules underpinning tissue structure and homeostasis, whereas molecules involved in immune cell recruitment and activation were expressed at lower levels compared to isotype antibody treatment (Extended Data Fig. 5e). Moreover, hearts from isotype-treated TCRM mice suffering from inflammatory dilated cardiomyopathy showed elevated COL1 expression in regions of myocardial damage (Extended Data Fig. 5f). Accordingly, staining for collagen networks in hearts of TCRM mice treated with 14-D10-2 and isotype control (Fig. 5g) and subsequent quantitative image analysis (Fig. 5h) revealed that anti-GREM1/2 treatment had significantly reduced the fibrotic remodeling of the myocardium. Anti-GREM1/2 treatment led to significantly improved cardiac function as determined by echocardiographic measurement of the ejection fraction in 8-week-old TCRM mice (Fig. 5i), which is most likely a result of the inflammation-attenuating and fibrosis-attenuating effect of BMP4 restoration. Notably, long-term treatment of mice with the anti-GREM1/2 antibody did not affect blood cell composition or the concentration of key serum enzymes that indicate liver or kidney damage, thus indicating no overt toxic effect of the treatment (Extended Data Fig. 5g). Overall, these data demonstrate that antibody-mediated GREM1/2 blockade efficiently ameliorates T cell-induced myocardial inflammation and loss of cardiomyocytes during the acute phase of myocarditis. In the long term, the treatment-induced restoration of BMP4 availability prevents progression to inflammatory cardiomyopathy through the reduction of fibrotic remodeling.

### Reduced BMP4 expression is a hallmark of human myocarditis

**Fig. 6:**
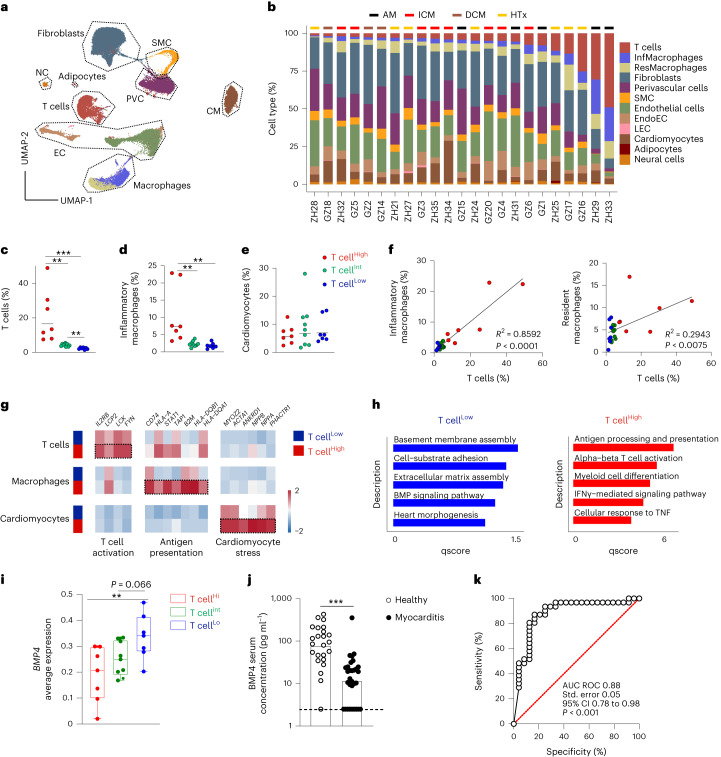
Regulation of BMP4 expression in human myocardial inflammatory disease. **a**–**k**, snRNA-seq from left or right ventricular EMBs from patients with acute myocarditis (AM) (*n* = 5), inflammatory cardiomyopathy (ICM) (*n* = 8), dilated cardiomyopathy (DCM) (*n* = 3) or undergoing heart transplantation (HTx) (*n* = 7). **a**,**b**, UMAP representation (**a**) and abundance of cardiac cell populations (**b**) in individual patients. **c**–**e**, Frequencies of T cells (**c**), inflammatory macrophages (**d**) and cardiomyocytes (**e**) in patients stratified according to high (>6%, T cell^High^, *n* = 7), intermediate (3–6%, T cell^Int^, *n* = 9) and low (<3%, T cell^Low^, *n* = 7) proportions of heart-infiltrating T cells. Dots indicate individual patients; bars indicate geometric means. **f**, Correlation between T cells and inflammatory macrophages (left) and T cells and resident macrophages (right). **g**, Heat maps showing average gene expression of the indicated, differentially expressed genes grouped by function in T cells, macrophages and cardiomyocytes. **h**, Significantly enriched pathways according to GO enrichment analysis based on differentially expressed genes between cardiac fibroblasts from T cell^Low^ and T cell^High^ cardiac biopsies. **i**, Average *BMP4* gene expression in cardiac fibroblast. Dots indicate values of individual patients; box plots as in Fig. 1. **j**, ELISA-based quantification of BMP4 concentration in serum from healthy donors or patients with biopsy-confirmed and/or cardiac MRI-confirmed acute myocarditis. Dots indicate values of individual patients; data are mean ± s.e.m. **k**, ROC curve of BMP4 serum concentrations of patients with acute myocarditis and healthy controls. snRNA-seq data represent a total of 44,114 nuclei, two biopsies per patient (*n* = 23 patients). Statistical analysis was performed using one-way ANOVA with Dunnett’s post test (**c**–**e**), Benjamini, Krieger and Yekutieli post test (**i**) or Mann–Whitney *U*-test (**k**) with **P < 0.01 and ***P < 0.001. Simple linear regression test was used in **f**. CI, confidence interval; LEC, lymphatic endothelial cells; NC, neural cells; SMC, smooth muscle cells. Source data

**Extended Data Fig. 6:**
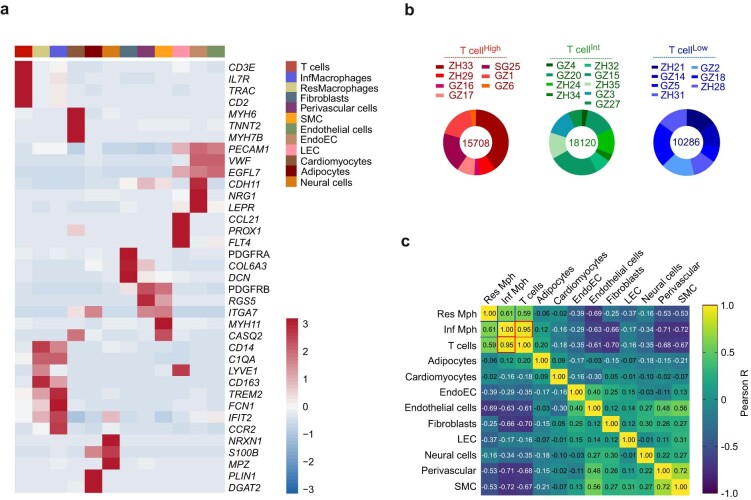
(**a**) Heatmap showing the expression of the marker genes used to assign the population identity of the different clusters obtained from snRNAseq analysis of endomyocardial biopsies (EMBs) of acute myocarditis, inflammatory cardiomyopathy, dilated cardiomyopathy or heart transplantation patients. (**b**) Pie charts depicting total number of nuclei from the different patients stratified by the percentage of infiltrating T cells with T cell^High^, T cell^Int^ and T cell^Low^ groups. (**c**) Correlation of multiple variables comparing the different proportions of cardiac cells in the snRNA-seq analysis of EMBs from 23 patients; Pearson’s R correlation values. Source data

**Extended Data Fig. 7:**
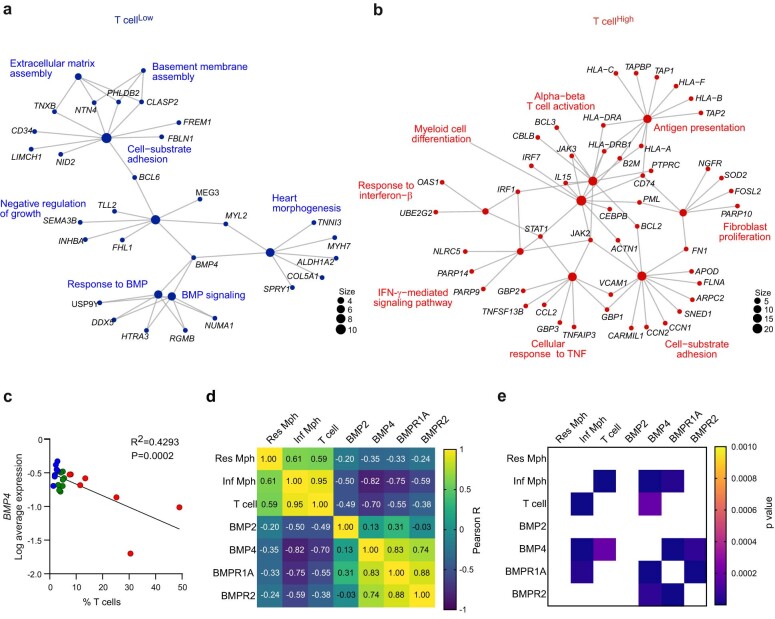
Network plots depicting significantly enriched gene sets in cardiac fibroblasts from (**a**) T cell^Low^ and (**b**) T cell^High^ groups according to GO enrichment analysis based on differentially expressed genes in snRNA-seq analysis of cardiac EMBs. (**c**) Correlation analysis BMP4 mRNA expression and T cell infiltration in EMBs. Colors indicate the proportion of infiltrating T cells. High, red dots (>6%, T cell^High^); intermediate, green dots (3–6%, T cell^Int^) and low, blue dots (<3%, T cell^Low^) proportions of heart-infiltrating T cells. (**d**, **e**) Multiparametric correlation analysis between proportions of infiltrating cardiac cells and genes of the BMP family. (d) Pearson’s correlation comparisons of indicated cell type abundances and expression of indicated genes. (e) P values from the Pearson’s correlation comparisons in (d). Statistical analysis was performed using simple linear regression test (c). Source data

To validate translational relevance for the observed dysregulated cardiac BMP activity during myocardial inflammation, we used snRNA-seq analysis to characterize the cellular and molecular landscape of inflamed human cardiac tissue. We analyzed endomyocardial biopsies (EMBs) from patients with suspected cardiac inflammation (acute myocarditis, inflammatory cardiomyopathy and dilated cardiomyopathy) as well as from patients undergoing routine EMB sampling after heart transplantation (Extended Data Table 1). Unbiased clustering analysis showed the different immune and non-hematopoietic cells (Fig. 6a) in the myocardium that could be distinguished by characteristic marker genes (Extended Data Fig. 6a). Cell composition analysis revealed the most striking changes in the abundance of T cells and inflammatory macrophages (Fig. 6b). Because T cell frequencies in healthy human heart tissue are lower than 3%^20,24^, we considered seven EMB samples (10,286 nuclei in total) as ‘T cell^Low^’ (Fig. 6c and Extended Data Fig. 6b). Seven EMB samples with the highest T cell content were assigned as ‘T cell^High^’ (15,708 nuclei), and EMB samples with intermediate T cell content between 3% and 7% were grouped as ‘T cell^Int^’ (18,120) (Fig. 6c and Extended Data Fig. 6b). The abundance of inflammatory macrophages was distributed in a similar fashion between the three groups (Fig. 6d), whereas the relative content of cardiomyocytes was not significantly different (Fig. 6e). Multiple-variable correlation analysis of cardiac cell abundances confirmed the strong correlation between T cells and the presence of inflammatory macrophages, whereas the accumulation of resident macrophages appeared to be less dependent on T cell infiltration (Fig. 6f and Extended Data Fig. 6c). Differential gene expression analysis of T cells, macrophages and cardiomyocytes from T cell^Low^ versus T cell^High^ EMBs showed an elevated activation status of both T cells and macrophages (Fig. 6g). Increased T cell abundance and activity in EMBs was associated with enhanced expression of genes that indicate cardiomyocyte stress and damage, such as *NPPA* and *NPPB*, which encode for the natriuretic peptides A and B (Fig. 6g). Gene set enrichment analyses of fibroblasts from T cell^Low^ and T cell^High^ EMBs confirmed that, also in the human myocardium, low abundance of T cells was associated with homeostatic fibroblast functions, including ECM assembly, heart morphogenesis and BMP signaling (Fig. 6h and Extended Data Fig. 7a). As expected, the high frequencies of T cells and macrophages in the T cell^High^ biopsies were associated with the rewiring of cardiac fibroblast function toward inflammatory processes, including antigen presentation, T cell activation and myeloid cell differentiation (Fig. 6h and Extended Data Fig. 7b). Correlation analysis across all EMB samples showed a negative and highly significant association between *BMP4* mRNA expression and T cell infiltration (Fig. 6i and Extended Data Fig. 7c). In addition, increased abundance of inflammatory, but not resident, macrophages was accompanied by downregulation of *BMP4* expression (Extended Data Fig. 7d,e). These data indicate that the global level of immune cell concentration and activation in the inflamed human myocardium is linked to dysregulated BMP4-mediated signaling.

Finally, we assessed whether BMP4 dysregulation in the inflamed myocardium is reflected by changes in BMP4 serum concentrations. To this end, we analyzed 31 serum samples from patients with EMB-confirmed and/or cardiac MRI-confirmed acute myocarditis (Extended Data Table 2) using a highly sensitive ELISA test. We found significantly reduced BMP4 serum concentration in patients with myocarditis in comparison to age-matched healthy volunteers (Fig. 6j). Moreover, the receiver operating characteristic (ROC) curve of BMP4 serum concentration indicated high sensitivity and specificity (Fig. 6k), highlighting that reduced BMP4 production is a major trait of perturbed myocardial homeostasis and a potential biomarker for the diagnosis in human myocarditis.

## Discussion

The functionality of multi-cellular tissues depends on the sustenance of homeostatic circuits that regulate cellular and molecular interactions within physiological concentrations and rates^35^. Inflammatory perturbation leads to loss of tissue structure and function and to overriding of homeostatic control mechanisms^36^. In the myocardium, various perturbations precipitate the activation of inflammatory pathways, such as virus-induced damage of cardiomyocytes^6^, activation of cardiomyocyte-specific T cells through bacterial mimic peptides^7^, impaired T cell control due to immune checkpoint inhibitor treatment^14^ or recognition of minor histocompatibility antigens by resident T cells in the cardiac microenvironment in the case of cardiac transplant rejection^37^. In the present study, we found that dysregulated BMP signaling in fibroblasts is a key factor for the maintenance of homeostatic circuits in the heart during T cell and macrophage-dominated myocardial inflammation. It appears that fibroblasts in both human and murine hearts are the main source of BMP4 and that BMP4 production by fibroblasts is significantly reduced in the cardiac microenvironment by inflammatory mediators, such as IL-1β. Notably, BMP4 downregulation and decreased signaling through the BMPR1A/BMPR2 complex are traits of the rapid phenotypical switch of cardiac fibroblasts from the homeostatic to the inflammatory state. Restoration of BMP4 availability in the myocardium through the application of an antibody that neutralizes the BMP4 inhibitors GREM1 and GREM2 reduced the extent of immunopathological damage through attenuated fibroblast activation, lowered myeloid and T cell recruitment and improved cardiomyocyte integrity. These findings highlight the key function of the BMP4–GREM1/2 axis for the maintenance of cardiac homeostasis and the control of myocardial inflammation.

Members of the BMP family play essential roles as morphogens during early embryonic development, for example through patterning of the dorsoventral body axis^38^ and during cardiac development as demonstrated for BMP2 and BMP4 (refs. ^39,40^). In the adult, BMP signaling is one of the pathways that appears to regulate cardiac remodeling^27–29^. Indeed, GREM2-dependent control of BMP2 availability affects the extent of immune cell infiltration and maintenance of heart function during experimental myocardial infarction in *Grem2*-deficient mice^41^. The multiple functions of BMP2 and BMP4 in cardiac development and integrity underscore the versatility and importance of this molecular circuit in cardiac homeostasis.

Pathways and factors that are crucial for balancing tissue homeostatis and inflammation need to be controlled on multiple levels. Because BMPs bind to their receptors as either homodimers or heterodimers, the combinatorial logic underlying BMP signaling is key for the understanding of context-specific functions and regulation of this pathway. For example, BMP2 and BMP4 display high ligand equivalence in terms of their ability to trigger BMPR signaling, which is indicative for a high system redundancy^42^. The stringent regulation of BMP2/4-mediated signals is secured by receptor antagonism of BMP10 (ref. ^42^) and through sequestering of BMP2 and BMP4 by the specific inhibitors GREM1 and GREM2 (ref. ^43^). Our finding that GREM1 and GREM2 concentration in the cardiac microenvironment did not significantly change during inflammatory processes indicates that BMP4 production by cardiac fibroblasts is controlled mainly on the transcriptional level. The use of the *Ccl19*-Cre mouse line in our study to elaborate BMP4-mediated functions supports the notion that specific subsets of cardiac fibroblasts acquire different functional states during inflammation. Further studies using cardiac fibroblast-specific Cre-driver mice will be required to fully dissect the role of the GREM1/2–BMP4 axis in myocardial homeostasis and inflammation. Clearly, antibody-mediated GREM1/2 inhibition increases BMP4 availability in the inflamed cardiac microenvironment—despite transcriptional downregulation in fibroblasts—and, thereby, ameliorates cardiac inflammation. Successful restoration of BMP4-mediated homeostatic functions of cardiac fibroblasts by treatment with anti-GREM1/2 antibody underscores the importance of the GREM1/2–BMP4 axis as a critical component within the regulatory networks that steer molecular and cellular processes in the heart.

Organ development and patterning of tissue microenvironments depend on temporal-spatial control of growth and differentiation factor production. BMPs, together with other homeostatic regulators, such as Wnt and Notch ligands, form gradients that regulate the epithelial stem cell niche of different tissues, such as the intestine^44^. In the adult cardiac microenvironment, BMPs most likely do not regulate the proliferation of the main functional cell type of this tissue—that is, the cardiomyocyte—because stemness and proliferative capacity of cardiomyocytes are lost in the neonatal phase^45^. However, the expression of the major BMP4 receptor by murine and human cardiomyocytes suggests that BMP signaling in adult cardiomyocytes controls processes other than proliferation. It will be important to determine in future work to what extent BMP signaling in cardiomyocytes affects homeostatic function within the perturbed tissue context.

Despite the complex biology of the BMP4–GREM1/2 axis with multiple redundancies and various layers of regulation, it is considered an interesting target for therapeutic intervention^43^. For example, it has been suggested to treat pulmonary arterial hypertension using antibody-mediated blockade of GREM1 (ref. ^46^). Moreover, recent work proposed to counter tumorigenesis in the intestine through the correction of aberrant epithelial GREM1 expression^47^ or to balance BMP signaling in stromal cells through selective GREM1 blockade^48^. The results of the present study indicate that the functional redundancy of GREM1 and GREM2, and other extracellular BMP inhibitors, should be taken into account for targeted manipulation of BMP signaling.

The development of treatment modalities for acute myocardial inflammation has received renewed attention with the initiation of randomized controlled trials assessing treatment of acute myocarditis with high-dose methylprednisolone or the interleukin-1 receptor antagonist anakinra^2^. The restoration of BMP4 availability in the inflamed cardiac microenvironment through pan-GREM blockade offers an alternative approach not only to achieve reduction of innate and/or adaptive immune responses but also to sustain homeostatic circuits that are key for the functioning of the heart. Thus, targeting of the BMP4–GREM1/2 axis in patients suffering from cardiac inflammation offers protection of cardiomyocyte integrity, reduction of cardiac fibrosis and, thereby, prevention of heart failure.

## Methods

Our research complies with all relevant ethical regulations for murine and human studies. The corresponding permission numbers for animal studies and the study protocols for human samples are indicated in their corresponding Methods subsection.

### Mice

MYH6-specific TCR transgenic mice (TCR-M) on the BALB/c background were previously described^30^. TCRM mice were maintained in heterozygous breeding, and transgene-negative littermates were used as controls. *Rag*^1*tm*1M*om*^*(Rag1*^−/−^) mice on the BALB/c background and C57BL/6 mice were obtained from The Jackson Laboratory. BAC-transgenic C57BL/6N-Tg (*Ccl19*-Cre)489Biat (*Ccl19*-Cre) mice crossed with R26R-EYFP mice were previously described^49^. To specifically ablate *Bmp4* expression in cardiac fibroblast, we crossed *Ccl19*-Cre R26R-EYFP mice with *Bmp4*^*fl/fl*^ mice (B6;129S4-Bmp4tm1Jfm/J, obtained from The Jackson Laboratory). All mice were maintained in individually ventilated cages. Experiments were performed in accordance with federal and cantonal guidelines (Tierschutzgesetz) under permission numbers SG02/19, SG07/20 and SG25/2020, after review and approval by the St. Gallen Cantonal Veterinary Office. Mice were treated intraperitonally twice per week with 200 µg of 14-D10-2 or 20-D1-5 or 3-A1-3 anti-GREM1/2 antibodies or IgG2b isotype control as indicated in the respective experiments.

### Myocarditis induction in *Rag1*^*−*^^/−^ mice

Spleens were collected from TCRM mice and disrupted on a 70-μm cell strainer. Red blood cells were lysed by osmotic shock, and 10^6^ splenocytes were injected intravenously in the lateral tail vein of *Rag1*^−/−^ mice. Fourteen days after adoptive transfer, mice were bled to confirm CD4^+^ T cell expansion. Disease activity scores and analysis of T cell activation in the heart were assessed at day 28 after adoptive transfer.

### Human endomyocardial biopsies, blood samples and clinical data

Two samples per patient were obtained from patients undergoing right or left ventricular EMBs as part of the clinical diagnostic routine. EMBs were snap frozen in liquid nitrogen and stored at −80 °C until analysis. The patients were enrolled in the following clinical protocols: ‘Deciphering the molecular landscape of human heart fibroblasts at the single-cell level—an exploratory research project (Heart-RNASeq-01)’, Cantonal Ethics Committee Zurich, permission 2020-0135; ‘Immunopathological pathways underlying myocarditis and inflammatory cardiomyopathy—an exploratory study (ImmpathCarditis)’, Cantonal Ethics Committee Zurich, permission 2021-01917; and Graz Endomyocardial Biopsy Registry, Ethics Committee of the Medical University of Graz, permission 32-575 ex 19/20 (Extended Data Table 1). Serum samples from healthy volunteers and patients with myocarditis enrolled in different clinical studies were analyzed: (1) ‘Immunopathological pathways underlying myocarditis and inflammatory cardiomyopathy—an exploratory study (ImmpathCarditis)’, Cantonal Ethics Committee Zurich, permission 2021-01917; (2) secondary investigation using patient samples collected from the MicroDCM cohort^7^, Ethics Committee of Eastern Switzerland, permission 2017-01853; and (3) Graz Endomyocardial Biopsy Registry, Ethics Committee of the Medical University of Graz, permission 32-575 ex 19/20 (Extended Data Table 2). All study participants provided written informed consent in accordance with the Declaration of Helsinki and the International Conference on Harmonization Guidelines for Good Clinical Practice. All regulations were followed according to the Austrian or Swiss authorities and according to the clinical protocols.

### Histopathological scoring of myocarditis and cardiac fibrosis

Histopathological analysis was performed as previously described^30^. In brief, hearts were fixed in 4% formaldehyde (Formafix) for at least 12 h and embedded in paraffin. Histopathological changes were evaluated after H&E staining. Myocarditis severity was evaluated using a semiquantitative scoring system: 0, no inflammation; 1, <100 inflammatory cells involved, small inflammatory lesions; 2, >100 inflammatory cells involved, larger inflammatory lesions; 3, >10% of the heart section involved in inflammation; 4, >30% of the heart section involved in inflammation; and 5, >30% of the heart section involved in inflammation with extensive fibrosis and dilation of ventricle. Images from heart sections were acquired using a Z1 Observer (Zeiss), and images were processed using ZEN 2.6 software (Zeiss).

Fibrosis was determined using a Picrosirius Red Stain Kit (Cardiac Muscle, Abcam) following the manufacturer’s instructions. Slides were scanned with a Pannoramic 250 Flash III scanner (3DHISTECH) at the Institute of Pathology, Kantonsspital St. Gallen, and the area of percentage of collagen networks per heart section was determined using QuPath 0.3.0 software^50^ by a blinded observer.

### Confocal microscopy

Tissues were processed for either vibratome or cryotome sectioning. For vibratome processing, tissues were fixed for 3–4 h at 4 °C in freshly prepared 4% paraformaldehyde (Merck Millipore) under agitation. Organs were embedded in 4% low-melting agarose (Invitrogen) in PBS and serially sectioned with a vibratome (Leica VT-1200). Next, 40-μm-thick sections were blocked in PBS containing 10% FCS, 1 mg ml^−1^ anti-Fcγ receptor (BD Biosciences) and 0.1% Triton X-100 (Sigma-Aldrich). For staining with anti-BMP4 antibody, hearts were shock frozen in an isopropanol–dry ice bath, embedded in FSC22 clear frozen section compound (Leica Biosystems) and sectioned by cryotome (Leica, CM1950). Then, 10-μm sections were mounted onto Superfrost PLUS slides (Epredia) and fixed for 30 min in methanol at −20 °C. Tissues were blocked in PBS containing 10% FCS, 1 mg ml^−1^ anti-Fcγ receptor (BD Biosciences) and 0.1% Triton X-100 (Sigma-Aldrich) and stained overnight at 4 °C with the indicated antibodies (Extended Data Table 3). Unconjugated and biotinylated antibodies were stained with the indicated secondary antibodies or streptavidin conjugates (Extended Data Table 3). Microscopy was performed using a confocal microscope (LSM 980, Zeiss), and images were recorded and processed with ZEN version 14.0.18.2010 software (Zeiss). Imaris version 9.2.1 (Oxford Instruments) was used for image analysis.

### Flow cytometry, cell sorting and SMAD1/5/9 phosphorylation assay

Euthanized mice were perfused with 20 ml of PBS before hearts were harvested. Hearts were minced into small pieces and placed into a six-well dish filled with RPMI 1640 medium containing 2% FCS, 20 mM HEPES (Lonza), 1 mg ml^−1^ collagenase P (Sigma-Aldrich) and 25 μg ml^−1^ DNase I (Applichem) and incubated at 37 °C under continuous stirring for 1 h. The remaining tissue pieces were mechanically disrupted. For immune cell analysis, mononuclear cells were enriched by centrifugation (25 min at 800*g*, 4 °C) on a 30–70% Percoll gradient (GE Healthcare). For fibroblast analysis, stromal cells were enriched by incubating the cell suspension with MACS anti-CD45 and anti-TER119 microbeads (Miltenyi Biotec) and passing through a MACS LS column (Miltenyi Biotec). The unbound single-cell suspension was stained for further flow cytometric analysis or used for cell sorting.

Single-cell suspensions were first stained with fixable Zombie Aqua viability dye (BioLegend) and incubated for 30 min on ice. After washing, cells were incubated for 20 min at 4 °C in PBS containing 2% FCS and 10 mM EDTA with fluorochrome-labeled antibodies (Extended Data Table 3).

To identify cardiac fibroblasts with active BMPR1A/BMPR2 signaling, cells were grouped by binarization according to a module score that was calculated based on the average mRNA expression of known downstream target genes. Cells with a positive module score were grouped as cells with active BMPR1A/BMPR2 signaling, and cells with a negative module score were grouped as cells with inactive BMPR1A/BMPR2 signaling. Computation of differentially expressed genes revealed significantly increased *Ncam1* (CD56) expression in cells with active versus inactive BMPR1A/BMPR2 signaling. Intracellular phospho-SMAD1/5/9 staining was performed by treatment of cells with Cytofix/Cytoperm Fixation and Permeabilization Solution (BD Biosciences) for 30 min, and subsequent staining was carried out with Phospho-SMAD1/5/9 antibody (Cell Signaling Technology) in Phosflow Perm/Wash Buffer I (BD Biosciences) as indicated in the manufacturer’s protocol. Cells were washed with Phosflow Perm/Wash Buffer I (BD Biosciences), resuspended in PBS containing 2% FCS and 2 mM EDTA and acquired with an LSRFortessa and FACSDiva (BD Biosciences, version 9.0.1) software. Analysis of pSMAD1/5/9 MFI was performed using FlowJo software version 10.6.2 (Tree Star). Mean fluorescence intensity (MFI) fold change in CD56^+^ fibroblasts was calculated relative to baseline pSMAD1/5/9 staining in CD56 fibroblasts. GraphPad Prism (version 8) was used for statistical analysis.

Cells were acquired with the LSRFortessa and analyzed using FlowJo version 10.6.2 software following established guidelines^51^. For cell sorting, a FACSMelody Cell Sorter (BD Biosciences) was used.

### Ex vivo restimulation and cytokine production assay

For assessment of ex vivo production of IFNγ and IL-17A by murine T cells, 10^6^ lymphocytes were incubated for 4 h at 37 °C in 96-well round-bottom plates in 200 µl of RPMI 5% FCS supplemented with 10 µg ml^−1^ Brefeldin A (Sigma-Aldrich). Cells were stimulated with 0.25 µg of the MYH6_614–629_ peptide (RSLKLMATLFSTYASADR, GenScript) or phorbol myristate acetate (50 ng ml^−1^, Sigma-Aldrich)/ionomycin (500 ng ml^−1^, Sigma-Aldrich) as positive control or were left untreated. After surface molecule labeling (CD45, CD3, CD4 and Vβ8), cells were stained with the fixable Zombie Aqua viability dye (BioLegend). Cells were fixed with Cytofix/Cytoperm (BD Biosciences) for 20 min. Fixed cells were incubated at 4 °C for 40 min with permeabilization buffer (2% FCS, 0.5% saponin in PBS) containing antibodies to IFNγ and IL-17A. Samples were measured using the LSRFortessa. Data were analyzed using FlowJo version 10.6.2 software.

### Isolation of nuclei from cardiac tissue

Single nuclei were obtained from shock-frozen murine or human cardiac tissues using the method described previously^20^ with minor modifications. In brief, frozen murine cardiac tissue samples (50–100 mg) or two EMBs from patients were put into 1 ml of ice-cold homogenization buffer (250 mM sucrose, 25 mM KCl, 5 mM MgCl_2_, 10 mM Tris-HCl, 1 mM dithiothreitol (DTT), 1× protease inhibitor, 0.4 U µl^−1^ RNase inhibitor, 0.1% Triton X-100 in nuclease-free water) and minced into small pieces using cold scissors (mouse tissue). Tissue pieces were gently minced using a plastic bar with the flat end. The homogenate was filtered through a 70-µm cell strainer (Corning), and remaining tissue was further minced with the plastic bar. The filter was washed twice with 1 ml of ice-cold homogenization buffer. The homogenate was further filtered through a 40-µm cell strainer (Corning), and the filter was washed twice with ice-cold homogenization buffer. Nuclei were centrifuged at 500*g* and 4 °C for 10 min, and the supernatant was removed and the pellet resuspended in storage buffer (1× PBS, 0.4 U µl^−1^ RNase inhibitor). Nuclei were stained with NucRed Live 647 (Invitrogen). Positive single nuclei were purified using the FACSMelody cell sorter (BD Biosciences) and visualized using FACSChorus (BD Biosciences, version 1.3).

### scRNA-seq and snNA-seq of murine samples

For scRNA-seq and snRNA-seq analyses, 23 samples were processed (BALB/c controls: four samples; TCRM: six samples; TCRM isotype antibody-treated: two samples; TCRM 14-D10-2-treated: two samples; BALB/c control fibroblasts: four samples; TCRM fibroblasts: five samples) and nine samples were processed for snRNA-seq analyses (BALB/c controls: two samples; TCRM cells: three samples; TCRM isotype antibody-treated: two samples; TCRM 14-D10-2-treated: two samples). Samples were processed and sequenced in five separate batches with all batches spanning multiple conditions. Single-cell/single-nucleus samples were run on a 10x Chromium analyzer (10x Genomics)^52^. cDNA library generation was performed following the established commercial protocol for Chromium Single Cell 3′ Reagent Kit (v3 chemistry). Libraries were run via NovaSeq 6000 for Illumina sequencing at the Functional Genomic Center Zurich. Gene expression was analyzed from sequencing data using Cell Ranger (version 5.0.1) ‘count’, with Ensembl GRCm38.9 as reference. Next, quality control was carried out in R (version 4.2.1) using the R/Bioconductor packages ‘scater’ (version 1.24.0)^53^ and SingleCellExperiment (version 1.18.0)^54^. This involved the identification and removal of damaged cells/nuclei or doublets, based on criteria including unusual unique molecular identifier (UMI) or gene counts (>2.5 median absolute deviation from the median across all cells) and high mitochondrial gene content (>2.5 median absolute deviations above the median across all cells). After performing quality control, the final dataset of total cells included 31,078 cells and 24,995 nuclei, and the dataset of sorted fibroblasts included 65,545 cells.

Downstream analysis was performed using the Seurat R package (version 4.1.1)^55^. For analysis of the cardiac cell dataset, all samples were merged and integrated across data using the IntegrateData function from the Seurat R package to account for differences between single-cell and single-nucleus data. Downstream analysis further included normalization, scaling, dimensional reduction with principal component analysis (PCA) and UMAP, graph-based clustering and calculation of unbiased cluster markers with dims = 1:20 PCA dimensions and a resolution of 0.25 as clustering parameters. Clusters were characterized based on the expression of calculated cluster markers and canonical marker genes as previously reported^56,57^. To examine expression signatures of fibroblasts in more detail, the fibroblast datasets with sorted fibroblast samples were merged and analyzed individually again using the Seurat R package for normalization, scaling, dimensional reduction with PCA and UMAP and graph-based clustering.

For comparative analysis between samples from TCRM versus control mice or isotype antibody-treated versus 14-D10-2 antibody-treated mice, differentially expressed genes were calculated using the FindAllMarkers function from the Seurat R package. Significantly enriched Gene Ontology (GO) terms were identified running the enrichGO function from the clusterProfiler R/Bioconductor package (version 4.0.5)^58^ on differentially expressed genes. Differentially expressed genes were further summarized in groups based on their known functions. The derived gene signatures were visualized using heat maps and by projecting their average expression on dimensional reduction plots, including UMAPs and diffusion maps. Diffusion maps were calculated using the DiffusionMap function from the ‘destiny’ R package (version 3.10.0)^59^.

### snRNA-seq of human heart biopsies

Sorted nuclei from human heart EMBs were run using the 10x Chromium (10x Genomics) system, and cDNA libraries were generated according to the established commercial protocols for Chromium Single Cell 3′ Reagent Kit (v3 chemistry) and Chromium Nuclei Isolation Kit. Libraries were sequenced by NovaSeq 6000 Illumina sequencing at the Functional Genomic Center Zurich, and gene expression was estimated using Cell Ranger (version 5.0.1) ‘count’, with Ensembl GRCh38.103 as reference. Quality control included the removal of nuclei with unusual UMI or gene counts (>2.5 median absolute deviation from the median across all cells) and was performed in R version 4.2.1 using the R/Bioconductor packages ‘scater’ (version 1.24.0)^53^ and SingleCellExperiment (version 1.18.0)^54^. After performing quality control, the final dataset included 44,114 nuclei from 23 EMBs.

For downstream analysis with the Seurat R package (version 4.3.0)^55^, all samples were merged and integrated across patient ID using the IntegrateData function. Integrated data were further processed running normalization, scaling, dimensional reduction with PCA and UMAP, graph-based clustering and calculation of unbiased cluster markers using dims = 1:20 PCA dimensions and a resolution of 0.6 as clustering parameters. Clusters were characterized based on the expression of calculated cluster markers and canonical marker genes as previously reported^19,20^. After cluster assignment, samples were grouped based on their T cell proportions, and groups were compared by calculating differentially expressed genes using the FindAllMarkers function from the Seurat R package. Finally, significantly enriched GO terms were identified by running the enrichGO function from the clusterProfiler R/Bioconductor package (version 4.4.4)^58^ on the differentially expressed genes.

### Interactome analysis

Interactome analysis was performed, inferring significant cell–cell communication using the R package CellChat^31^. To account for differences in cell type abundances, the communication probability/strength among all interacting cell types was calculated using the computeCommunProb function with population.size = TRUE and the default ‘triMean’ method for the calculation of the average gene expression per cell group. Significant alterations in outgoing and incoming signaling between the two conditions were determined and visualized using the netAnalysis_signalingRole_scatter function. Differential signaling in fibroblasts between conditions was further examined by calculating the information flow of each ligand–receptor pair in the control and TCRM mice, respectively. Information flow of a ligand–receptor pair is obtained by summing the communication probability (interaction strength) among all pairs of cell groups in the inferred network for the specific ligand–receptor pair. Significant differences in the information flow were visualized using the rankNet function.

### RNA isolation and RT–PCR analysis

RNA was isolated from sorted cells using an RNeasy Mini Kit (Qiagen). Contaminating DNA was eliminated through on-column DNase digestion (Zymo Research). To generate cDNA for RT–PCR analysis, we used a High-Capacity cDNA Reverse Transcription Kit (Applied Biosystems). RT–PCR amplification and quantification were performed using a real-time PCR machine (QuantStudio 5, Thermo Fisher Scientific). Amplification was done using a LightCycler FastStart DNA Master SYBR Green I Kit (Roche), and Hprt was used as housekeeping gene. Reactions were performed in duplicate, and relative gene expression was calculated using the ΔΔCt method. The following primers were used: *Bmp4*, QT00111174; *Bmp2*, QT01054277; *Ccl2*, QT00167832; *Il6*, QT00098875; *Tnf*, QT00104006; and *Hprt*, QT00166768 (Qiagen).

### Generation of anti-GREM1/2 antibodies

C57BL/6 mice were immunized with 10 μg of human GREM1 (PeproTech) conjugated with KLH (Thermo Fisher Scientific) and emulsified in Complete Freund’s Adjuvant (CFA, BD Biosciences) on days 0, 15 and 70 (subcutaneously) and on day 90 (intravenously without CFA). On day 97, splenocytes were obtained and fused with P3x63Ag8.653 myeloma cells. Hybridoma cells were grown in IMDM selective media (Gibco) supplemented with 10% FCS (Lonza), 0.05 mM mercaptoethanol (Gibco), 100 IU ml^−1^ penicillin–streptomycin (Lonza), 0.8 μg of recombinant mouse IL-6 (PeproTech) and 1× hypoxanthine–aminopterin–thymidine (Sigma-Aldrich). Supernatant of wells containing growing cells was screened for specific antibodies against HumGREM1 by ELISA. After expansion of GREM1-specific hybridomas, a limiting dilution step was performed. Monoclonal hybridomas were expanded and used for antibody purification. Antibodies were purified from the supernatant using a HiTrap Protein G affinity column pre-equilibrated in binding buffer (20 mM sodium phosphate, pH 7.0) and then washed with binding buffer and eluted with elution buffer (0.1 M glycine-HCl, pH 2.7). Purified antibodies were collected in fractions of 10 ml into 5 ml of PBS and 100 μl of neutralization buffer (1 M Tris-HCl, pH 9.0), and buffer exchange was performed overnight using 10-kDa dialysis cassettes (Thermo Fisher Scientific).

### BMP4 and IL-1β ELISA

For the assessment of BMP4 and IL-1b concentration in tissue, hearts from TCRM or control mice were isolated after complete perfusion using cold PBS. The hearts were weighted and frozen in PBS at −80 °C until analysis. Homogenates were generated using a MagNA Lyser (Roche) using two cycles of 5,000 r.p.m., 50 s. The tubes were placed immediately on ice after homogenization. The samples were centrifuged at 2,500 r.p.m., 5 min, 4 °C, and the supernatant was used to perform the ELISA measurements. BMP4 and IL-1β concentrations were determined following the manufacturer’s instructions (RayBiotech and Abcam, respectively).

Serum from healthy donors or patients with myocarditis were taken by venipuncture; sera was separated by centrifugation; and samples were preserved at −20 °C until analysis. BMP4 levels in the samples were measured using a human BMP4 SimpleStep ELISA Kit (Abcam) following the manufacturer’s instructions. All samples were diluted 1:4 using Sample Diluent NS and tested in duplicates. Values per patient are displayed as the mean value from duplicates. The detection limit of the assay is 2.5 pg ml^−1^.

### GREM1 and GREM2 ELISA

An ELISA was used to analyze the anti-GREM1 antibodies for binding against GREM1 and GREM2. High-binding 96-well polystyrene plates (Corning) were coated with GREM1 or GREM2 conjugated to BSA in 0.1 M carbonate-bicarbonate buffer, pH 9.5. Plates were incubated overnight at 4 °C. The plates were washed four times with PBS containing 0.05% Tween 20 (PBS-T) (Sigma-Aldrich) and blocked with 5% non-fat dry milk diluted in PBS (PBS-M) for 1 h at 37 °C. After washing, different concentrations of the monolonal antibodies were diluted in PBS-M and added to the wells. Plates were incubated for 1 h at 37 °C, followed by four washes with PBS-T and then incubated for 1 h of at 37 °C with HRP-conjugated goat-anti-mouse IgG antibody (1:1,000 in PBS-M, Jackson ImmunoResearch). After four washes with PBS-T, ortho-phenylenediamine (0.5 mg ml^−1^, Sigma-Aldrich) in 0.1 M citrate buffer, pH 5.6, containing 0.08% H_2_O_2_ was used to develop the reaction, and the reaction was stopped after 10 min by adding 2.5 N sulfuric acid. Optical density was measured at 492 nm using an automated ELISA plate reader (Tecan). Binding avidity of the antibodies was determined using serial dilutions of the recombinant human GREM1 or GREM2 and of the respective antibody and calculated as the area under the curve (AUC) using GraphPad Prism 8.0.

### GREM1 and GREM2 neutralization assay

The SL-0051 cell line (Signosis) was used to determine in vitro BMP4 activity and to assess the neutralization activity of anti-GREM1/2 antibodies. SL-0051 cells are a HEK293 cell line stably transfected with a luciferase reporter based on a pTA-BMP luciferase reporter vector that contains four repeats of BMP binding sites and a minimal promoter upstream of the firefly luciferase coding region. SL-0051 cells were grown in DMEM medium supplemented with 10% FCS. Cells were seeded in 96-well flat-bottom plates at a concentration of 1.5 × 10^4^ cells in 100 μl per well and incubated overnight at 37 °C. On the following day, 50 μl of medium was gently removed from each well. In a separate 96-well round-bottom plate, each antibody was serially diluted 1:2 and tested in triplicates. Recombinant human GREM1 or GREM2 was added to the plate at different concentrations. This mixture was transferred to the plate containing SL-0051 cells and incubated for 20 min at 37 °C. After the incubation, 50 µl per well of recombinant human BMP4 was added at a concentration of 0.5 μg ml^−1^. The plates were incubated for 24 h at 37 °C. After incubation, the medium was gently flicked off, and cells were washed twice with 200 μl of PBS, and then 20 µl of lysis buffer was added and incubated for 30 min at room temperature. Next, 100 µl per well of Luciferase Substrate (Signosis, P/N LUC015) was added, and the chemoluminescence was measured in a Luminometer (TECAN). EC_50_ was calculated in GraphPad Prism 8.0 from dose–response curves using relative BMP4 activity values.

### Isolation of cardiac fibroblasts and in vitro stimulation

Cardiac fibroblasts were isolated from hearts of 8-week-old *Ccl19*-Cre R26R-EYFP mice. Sorted EYFP^+^, EYFP^−^ or bulk cardiac fibroblasts were cultured for 10 days in RPMI 1640/10% FCS, 1% penicillin–streptomycin (Sigma-Aldrich) and 16 µg ml^−1^ gentamicin. In total, 10,000 cells were stimulated with BMP4 (10 ng ml^−1^, R&D Systems) or IL-1β (1 ng ml^−1^, Thermo Fisher Scientific) or left untreated (medium). Supernatants were collected after 24 h, and BMP2 (Abcam), BMP4 (RayBiotech), BMP7 (Abcam) and BMP9 (Abcam) concentrations were determined by ELISA. IL-6 and TNF concentrations were determined using cytometric bead array (BD Biosciences). Cells were detached using trypsin-EDTA 0.25% (Gibco) and incubated with anti-ICAM1 (CD54) and Fixable Viability Stain 510 (1:1,000, BD Biosciences). Data were acquired with the LSRFortessa and FACSDiva (version 9.0.1) software for cell counting, and ICAM1 MFI analysis was performed using FlowJo version 10.6.2 software. For confocal microscopy analysis, 5,000 cardiac fibroblasts were seeded in cell culture chamber slides (Thermo Fisher Scientific) and left for 24 h to attach. Slides were fixed for 20 min using 4% paraformaldehyde (Merck Millipore) in PBS. Fixed slides were further washed with PBS containing 1% Triton X-100 (Sigma-Aldrich) and 2% FCS (Sigma-Aldrich) for 1 h at 4 °C. Slides were further incubated with anti-COL1 (Sigma-Aldrich), anti-SMA (Sigma-Aldrich) and anti-EYFP (Clontech). Unconjugated antibodies were detected with the following secondary antibodies: Dy Light 649-conjugated anti-rat IgG and Alexa Fluor 488-conjugated anti-rabbit IgG (all purchased from Jackson Immunotools). Microscopy was performed using a confocal microscope (LSM 980, Zeiss), and images were recorded and processed with ZEN 2010 software (Zeiss) and Imaris version 9 (Bitplane).

### Clinical chemistry

Blood was harvested and collected either in EDTA tubes (Microvette) for complete blood cell count assay or in serum collection tubes (Microvette) for biochemical analyses (aspartate aminotransferase (AST), alanine transaminase (ALT) and creatine kinase (CK)). Blood samples were analyzed by the Veterinary Laboratory of the University of Zurich using a Sysmex XN-1000 analyzer (Sysmex) for blood cell analysis and a Cobas 6000 analyzer (Roche) for clinical chemistry.

### Echocardiography of mice

Anesthesia was induced in mice using 3–4% isofluorane (Attane, Provet) and compressed standard breathing air. As soon as the hind limb reflex became undetectable, mice were placed on a heating plate (41.5 °C) in supine position while still under continuous isofluorane (1–2%) exposure. Vital parameters were continuously monitored through echocardiography, and body temperature was measured via rectal temperature probe. After chemical hair removal (Veet PURE), echocardiography sequences were acquired using B-mode and M-mode in parasternal long axis or parasternal short axis using a VisualSonics Vevo 3100 (Fujifilm) and analyzed using Vevo LAB version 5.7.1 software (Fujifilm).

### Statistical analysis

Statistical analyses were performed with GraphPad Prism 8.0 using unpaired two-tailed Student’s *t*-test or Mann–Whitney *U*-test. Longitudinal comparison between different groups was performed by one-way ANOVA with Tukey’s post test or two-way ANOVA with Bonferroni’s post test or Kruskall–Wallis *H*-test for non-parametric data. Statistical significance was defined as *P* < 0.05.

### Reporting summary

Further information on research design is available in the Nature Portfolio Reporting Summary linked to this article.

### Supplementary information

Reporting Summary

Supplementary Table 1 Differential gene expression for marker genes used to define the clusters in Fig. 1f

### Source data

Source Data Fig. 1 Statistical Source Data

Source Data Fig. 2 Statistical Source Data

Source Data Fig. 3 Statistical Source Data

Source Data Fig. 4 Statistical Source Data

Source Data Fig. 5 Statistical Source Data

Source Data Fig. 6 Statistical Source Data

Source Data Extended Fig. 1 Statistical Source Data

Source Data Extended Fig. 2 Statistical Source Data

Source Data Extended Fig. 3 Statistical Source Data

Source Data Extended Fig. 4 Statistical Source Data

Source Data Extended Fig. 5 Statistical Source Data

Source Data Extended Fig. 6 Statistical Source Data

Source Data Extended Fig. 7 Statistical Source Data

## Data Availability

The scRNA-seq and snRNA-seq data generated in this study have been deposited in the BioStudies database (https://www.ebi.ac.uk/biostudies/). The mouse data are available under accession numbers E-MTAB-12589 and E-MTAB-12559; the human data are available under accession number E-MTAB-12584. The processed data files can be downloaded from the Figshare platform at 10.6084/m9.figshare.24994478 and explored via an interactive browser at https://immbiosg.github.io/FRCdataExplorer/. All other data supporting the findings in this study are included in the main article and associated files.

## Extended data

**Extended Data Table 1:** Main clinical diagnosis and patient characteristics of the EMB cohort

	Acute myocarditis^a^ (*n* = 5)	Inflammatory cardiomyopathy^a^ (*n* = 8)	Heart transplantation^b^ (*n* = 7)	Dilated cardiomyopathy^a^ (*n* = 3)	Total (*n* = 23)
Sex					
Female (%)	2 (40.0%)	1 (12.5%)	2 (28.6%)	1 (33.3%)	6 (26.1%)
Male (%)	3 (60.0%)	7 (87.5%)	5 (71.4%)	2 (66.7%)	17 (73.9%)
Age (years)					
Mean (s.d.)	42.0 (18.07)	54.8 (14.67)	55.0 (9.66)	56.0 (5.20)	52.2 (13.6)
Median (minimum, maximum)	34.0 (27.0, 70.0)	56.5 (32.0, 76.0)	57.0 (34.0, 62.0)	59.0 (59.0, 50.0)	57.0 (27.0, 76.0)
Recruiting center ^**c**^					
Zurich (%)	3 (60.0%)	3 (37.5%)	5 (71.4%)	0 (0.0%)	11 (47.8%)
Graz (%)	2 (40.0%)	5 (62.5%)	2 (28.6%)	3 (100.0%)	12 (52.2%)

^a^ Main clinical diagnosis as documented in the patient information system.

^b^ EMBs were obtained as part of the routine clinical follow-up of heart transplant patients.

^c^ Recruiting centers: Clinic for Cardiology, University Hospital Zurich, Zurich, Switzerland; Division of Cardiology, University Hospital Graz, Graz, Austria.

Main clinical diagnosis and characteristics from patients with acute myocarditis (*n* = 5), inflammatory cardiomyopathy (*n* = 8), dilated cardiomyopathy (*n* = 3) or undergoing heart transplantation (*n* = 7) who underwent EMB.

**Extended Data Table 2:** Demographic details of patients with myocarditis and healthy volunteers

	*n*	Sex (M/F)	Age ± s.d.
Myocarditis ^a^	31	26/5	33.9 ± 14.3
Healthy	24	13/11	35.9 ± 9.2

^a^ Patients with myocarditis from the following cohorts—MicroDCM, St. Gallen (*n* = 21), ImmpathCarditis, Zurich (*n* = 8), Graz (*n* = 2)—with cardiological diagnosis confirmed by cardiac magnetic resonance imaging and/or EMB.

Demographics of patients with myocarditis and healthy volunteers with ELISA-based quantification of serum BMP4 concentration.

**Extended Data Table 3:** Antibodies used in this study

Clone	Reagent	Conjugate	Concentration (µg ml^−1^)	Source
IA8	anti-Ly6G	PE	1	Pharmingen
AL-21	anti-Ly6C	PerCP Cy5	2	BD Biosciences
145-2C11	anti-CD3e	PerCP	5	BioLegend
30-F11	anti-CD45	APC-Cy7	2	BioLegend
30-F11	anti-CD45	PE	2	BioLegend
B20.1	anti-Vα2 TCR	APC	2	BD Biosciences
MR5-2	anti-Vβ8.1/8.2 TCR	FITC	5	BD Biosciences
RM4-5	anti-CD4	BV-605	2	BioLegend
XMG1.2	anti-IFNγ	APC	2	BioLegend
TC11-18H10.1	anti-IL17	PE	2	BioLegend
2G9	anti-IA/IE	FITC	2	BD Biosciences
SA011F11	anti-CX3CR1	APC-Fire750	2	BioLegend
SA203G11	anti-CCR2	BV-421	2	BioLegend
X54-5/7.1	anti-CD64	PeCy7	0.8	BioLegend
MEC13.3	anti-CD31	A647	2	BioLegend
MEC14.7	anti-CD34	BV421	2	BioLegend
104	anti-CD45.2	BV510	2	BioLegend
AMS-32.1	anti-IA^d^	PE	1	BD Biosciences
3E2	anti-ICAM-1/CD54	BV421	2	BD Biosciences
APA5	anti-PDGFRα/CD140α	BV605	2	BD Biosciences
BP3	CD157/BST1	PE	2	BioLegend
8.1.1	anti-PDPN	PeCy7	2	BioLegend
563260	streptavidin	BV605	2	BD Biosciences
D7	anti-Sca-1/Ly6A/E	PerCP	2	BioLegend
RM-45	anti-CD4	Alexa 488	1	BioLegend
RAM-34	anti-CD34	Biotin	1	Invitrogen
M1/70	anti-CD11b	Alexa 647	1	BioLegend
30-F11	anti-CD45	Alexa 488		BioLegend
Ab155033	rabbit anti-BMP4	None	1	Abcam
AB-758	goat anti-COL1	None	0.8	Merck
711-165-152	donkey anti-rabbit	Cy3	1	Jackson ImmunoResearch
705-605-147	donkey anti-goat	Alexa 647	1	Jackson ImmunoResearch
016-600-984	streptavidin	Alexa 647	1	Jackson ImmunoResearch
TER-119	anti-Ter119	BV510	2	BioLegend
MVCAM.A	anti-VCAM-1/CD106	Biotin	2	BioLegend
Monoclonal antibody 13820	anti-pSMAD1/5/9	PE	1	Cell Signaling Technology

Antibodies used in this study for confocal microscopy and flow cytometry.

## Code availability

The code used for data analysis in this project is available at GitHub (mouse data: https://github.com/mluetge/Myocardial_Inflammation_mouse; human data: https://github.com/mluetge/Myocardial_Inflammation_human).
