# Supplementary material for: Bone morphogenic protein-4 availability in the cardiac microenvironment controls inflammation and fibrosis in autoimmune myocarditis
Source: Nat Cardiovasc Res. 2024 Feb 19;3(3):301–16. doi: 10.1038/s44161-024-00432-0 (PMC11358008; doi:10.1038/s44161-024-00432-0)
Supplement: Supplementary file 1 — Reporting Summary [file 44161_2024_432_MOESM1_ESM.pdf]

nature portfolio

Corresponding author(s): Burkhard Ludewig

Last updated by author(s): 2024/01/10

Reporting Summary

Nature Portfolio wishes to improve the reproducibility of the work that we publish. This form provides structure for consistency and transparency in reporting. For further information on Nature Portfolio policies, see our Editorial Policies and the Editorial Policy Checklist.

Statistics

For all statistical analyses, confirm that the following items are present in the figure legend, table legend, main text, or Methods section.

n/a	Confirmed	
☐	☒	The exact sample size (*n*) for each experimental group/condition, given as a discrete number and unit of measurement
☐	☒	A statement on whether measurements were taken from distinct samples or whether the same sample was measured repeatedly
☐	☒	The statistical test(s) used AND whether they are one- or two-sided<br>*Only common tests should be described solely by name; describe more complex techniques in the Methods section.*
☐	☒	A description of all covariates tested
☐	☒	A description of any assumptions or corrections, such as tests of normality and adjustment for multiple comparisons
☐	☒	A full description of the statistical parameters including central tendency (e.g. means) or other basic estimates (e.g. regression coefficient) AND variation (e.g. standard deviation) or associated estimates of uncertainty (e.g. confidence intervals)
☐	☒	For null hypothesis testing, the test statistic (e.g. *F*, *t*, *r*) with confidence intervals, effect sizes, degrees of freedom and *P* value noted<br>*Give P values as exact values whenever suitable.*
☒	☐	For Bayesian analysis, information on the choice of priors and Markov chain Monte Carlo settings
☒	☐	For hierarchical and complex designs, identification of the appropriate level for tests and full reporting of outcomes
☐	☒	Estimates of effect sizes (e.g. Cohen's *d*, Pearson's *r*), indicating how they were calculated

Our web collection on statistics for biologists contains articles on many of the points above.

Software and code

Policy information about availability of computer code

Data collection	FACSDiva (BD Biosciences v9.0.1), FACSChorus (BD Biosciences, v1.3), ZEN (Zeiss, V2010, v14.0.18.2010, V2.6), Visualsonics Vevo 3100 (Fujifilm)
Data analysis	CellChat (v1.1.3), Imaris (v9), Prism (Graphpad v8), FlowJo (Tree Star Inc., v10.6.2), R (v.4.2.1), CellRanger (v3.0.2), R/Bioconductor package scater (v.1.16.0), Seurat R package (v.4.1.1, v4.3.0), clusterProfiler R/Bioconductor (v.4.4.4, v4.0.5), Qpath0.3.0 software, VevoLABb Software Version 5.7.1 (Fujifilm).

For manuscripts utilizing custom algorithms or software that are central to the research but not yet described in published literature, software must be made available to editors and reviewers. We strongly encourage code deposition in a community repository (e.g. GitHub). See the Nature Portfolio guidelines for submitting code & software for further information.

Data

Policy information about availability of data

All manuscripts must include a data availability statement. This statement should provide the following information, where applicable:

- Accession codes, unique identifiers, or web links for publicly available datasets
- A description of any restrictions on data availability
- For clinical datasets or third party data, please ensure that the statement adheres to our policy

The single cell and single nucleus RNA-seq data generated in this study has been deposited in the BioStudies database (www.ebi.ac.uk/biostudies/) under accession

numbers E-MTAB-12584, E-MTAB-12589, E-MTAB-12559. Exploration of the transcriptomics data from this study is feasible on the interactive browser at https://immbiosg.github.io/FRCdataExplorer/.

Human research participants

Policy information about studies involving human research participants and Sex and Gender in Research.

Reporting on sex and gender	Patients from both sexes were recruited. No sex-based analyses have been performed due to the explortory nature of the study.
Population characteristics	Detailed information is listed in extended data tables 1 and 2
Recruitment	Patients with with suspected cardiac inflammation: acute myocarditis (AM), inflammatory cardiomyopathy (ICM), dilated cardiomyopathy (DCM), as well as patients undergoing EMB sampling after heart transplantation (HTx).
Ethics oversight	Cantonal Ethics Committee Zurich; Graz Endomyocardial Biopsy Registry, Ethics committee of the Medical University of Graz; Ethics Committee of Eastern Switzerland.

Note that full information on the approval of the study protocol must also be provided in the manuscript.

Field-specific reporting

Please select the one below that is the best fit for your research. If you are not sure, read the appropriate sections before making your selection.

☒ Life sciences ☐ Behavioural & social sciences ☐ Ecological, evolutionary & environmental sciences

For a reference copy of the document with all sections, see nature.com/documents/nr-reporting-summary-flat.pdf

Life sciences study design

All studies must disclose on these points even when the disclosure is negative.

Sample size	No sample-size calculation was performed. Sample sizes were determined to be adequate based on the reproducibility between independent experiments and patients based on previous studies from our laboratory.
Data exclusions	No data points were excluded.
Replication	For analysis of the performed scRNA-seq experiments no batch correction needed to be applied for any of the samples. Therefore we can assume careful and good reproducibility.
Randomization	Mice were randomly allocated to the different experimental groups. Randomization and control of covariant was not relevant in the allocation of the patients as all patients were grouped based on the results of the sn-RNAseq analysis
Blinding	Blinding was not performed since data analysis was explorative. No blinding was relevant for this study as patients were grouped after sn-RNAseq analysis regardless the etiology of the cardiac inflammation.

Reporting for specific materials, systems and methods

We require information from authors about some types of materials, experimental systems and methods used in many studies. Here, indicate whether each material, system or method listed is relevant to your study. If you are not sure if a list item applies to your research, read the appropriate section before selecting a response.

Materials & experimental systems

n/a	Involved in the study	
☐	☒	Antibodies
☐	☒	Eukaryotic cell lines
☒	☐	Palaeontology and archaeology
☐	☒	Animals and other organisms
☐	☒	Clinical data
☒	☐	Dual use research of concern

Methods

n/a	Involved in the study	
☒	☐	ChIP-seq
☐	☒	Flow cytometry
☒	☐	MRI-based neuroimaging

Antibodies

Antibodies used

Clone	Reagent	Conjugate	Concentration (µg/ml)	Source
IA8	anti-Ly6G	PE	1	Pharmigen
AL-21	anti-Ly6C	PerCP Cy5	2	BDBioscience
145-2C11	anti-CD3e	PerCP	5	Biolegend
30-F11	anti-CD45	APC-Cy7	2	Biolegend
30-F11	anti-CD45	PE	2	Biolegend
B20.1	anti-Vα2 TCR	APC	2	BDBioscience
MR5-2	anti-Vβ8.1/8.2 TCR	FITC	5	BDBioscience
RM4-5	anti-CD4	BV-605	2	Biolegend
XMG1.2	anti-IFN□	APC	2	Biolegend
TC11-18H10.1	anti-IL17	PE	2	Biolegend
2G9	anti-IA/IE	FITC	2	BDBioscience
SA011F11	anti-CX3CR1	APC-Fire750	2	Biolegend
SA203G11	anti-CCR2	BV-421	2	Biolegend
X54-5/7.1	anti-CD64	PeCy7	0.8	Biolegend
MEC13.3	anti-CD31	A647	2	Biolegend
MEC14.7	anti-CD34	BV421	2	Biolegend
104	anti-CD45.2	BV510	2	Biolegend
AMS-32.1	anti-IAd	PE	1	BDBioscience
3E2	anti-ICAM-1/ CD54	BV421	2	BDBioscience
APA5	anti-PDGFRα/ CD140α	BV605	2	BDBioscience
BP3	CD157/BST1	PE	2	Biolegend
8.1.1	anti-PDPN	PeCy7	2	Biolegend
563260	Streptavidin	BV605	2	BDBioscience
D7	anti-Sca-1/ Ly6A/E	PerCP	2	Biolegend
RM-45	Anti-CD4	Alexa 488	1	Biolegend
RAM-34	Anti-CD34	Biotin	1	Invitrogen
M1/70	Anti-CD11b	Alexa 647	1	Biolegend
30-F11	Anti-CD45	Alexa 488		Biolegend
Ab155033	Rabbit anti-BMP4	None	1	Abcam
AB-758	Goat anti-COL1	None	0.8	Merck
711-165-152	Donkey anti-rabbit	Cy3	1	JacksonImmunoresearch
705-605-147	Donkey anti-goat	Alexa 647	1	JacksonImmunoresearch
016-600-984	Streptavidin	Alexa 647	1	JacksonImmunoresearch
TER-119	anti-Ter119	BV510	2	Biolegend
MVCAM.A	anti-VCAM-1/ CD106	Biotin	2	Biolegend
mAb #13820	anti-pSMAD1/5/9	PE	1	Cell Signaling Technology, Inc.

Validation

All antibodies with the exception of anti-Gremlin1/2 clones 14-D10-2, 20-D1-5, 3-A1-3, came from commercial vendors, and were validated by the manufacturers on their official website.
The following antibodies were validated to bind murine cell and used for FlowCytometric analysis by the manufacturer: A8 anti-Ly6G PE 1 Pharmingen, AL-21 anti-Ly6C PerCP Cy5 2 BDBioscience, 145-2C11 anti-CD3e PerCP 5 Biolegend, 30-F11 anti-CD45 APC-Cy7 2 Biolegend
30-F11 anti-CD45 PE 2 Biolegend B20.1 anti-Vα2 TCR APC 2 BDBioscience, MR5-2 anti-Vβ8.1/8.2 TCR FITC 5 BDBioscience, RM4-5 anti-CD4 BV-605 2 Biolegend,XMG1.2 anti-IFN□ APC 2 Biolegend, TC11-18H10.1 anti-IL17 PE 2 Biolegend, 2G9 anti-IA/IE FITC 2 BDBioscience, SA011F11 anti-CX3CR1 APC-Fire750 2 Biolegend, SA203G11 anti-CCR2 BV-421 2 Biolegend, X54-5/7.1 anti-CD64 PeCy7 0.8 Biolegend, MEC13.3 anti-CD31 A647 2 Biolegend, MEC14.7 anti-CD34 BV421 2 Biolegend, 104 anti-CD45.2 BV510 2 Biolegend, AMS-32.1 anti-IAd PE 1 BDBioscience
3E2 anti-ICAM-1/ CD54 BV421 2 BDBioscience, APA5 anti-PDGFRα/ CD140α BV605 2
BDBioscience, BP3 CD157/BST1 PE 2 Biolegend, 8.1.1 anti-PDPN PeCy7 2
Biolegend, 563260 Streptavidin BV605 2 BDBioscience, D7 anti-Sca-1/ Ly6A/E PerCP 2
Biolegend, RM-45 Anti-CD4 Alexa 488 1 Biolegend, M1/70 Anti-CD11b Alexa 647 1
Biolegend, 30-F11 Anti-CD45 Alexa 488 Biolegend, Ab155033 Rabbit anti-BMP4 None 1 Abcam,
mAb #13820 anti-pSMAD1/5/9 PE 1 Cell Signaling Technology, Inc.
Anti- human mouse/human Gremlin-1/2 Clones: 14-D10-2, 20-D1-5, 3-A1-3. Generated for this study at the Kantonsspital St.Gallen, St. Gallen, Switzerland. See supplementary figure 3.

Eukaryotic cell lines

Policy information about cell lines and Sex and Gender in Research

Cell line source(s)

SMAD/BMP Responsive Luciferase Reporter HEK293 Stable Cell Line SL-0051; SKU: SL-0051 (Signosis, USA)

Authentication

Signosis has established BMP luciferase reporter Hek293 stable cell line that has been stably transfected with pTA-BMP-luciferase reporter vector, which contains 4 repeats of BMP binding sites, a minimal promoter upstream of the firefly luciferase coding region. Therefore, the cell line can be used as a reporter system for monitoring the activation of BMP triggered by stimuli treatment, enforced gene expression and gene knockdown.
Principle behind TF luciferase reporter. TF luciferase reporter stable cell line utilizes artificial promoter constructs to drive

	luciferase expression. The promoter region can consists of multiple repeats of a cis-element TF binding site, a DNA fragment from the promoter region of a known TF downstream gene, or a DNA fragment containing putative/known TF binding sites. There are several ways that a TF can be activated, such as through extracellular stimuli or through intracellular signaling pathways. Once activated, the TF translocates to the nucleus and often interacts with relevant co-factors to drive gene expression. Once luciferase is expressed, it can generate light in an enzymatic assay and the amount of light measured is positively correlated with the level of TF activation.
Mycoplasma contamination	Cell were not tested for Mycoplasma
Commonly misidentified lines (See ICLAC register)	No commonly misidentified cell lines were used in the study

Animals and other research organisms

Policy information about studies involving animals; ARRIVE guidelines recommended for reporting animal research, and Sex and Gender in Research

Laboratory animals	MYH6-specific TCR transgenic mice (TCR-M) on the Balb/c background have been previously described . TCRM mice were maintained in heterozygous breeding and transgene-negative littermates were used as controls. Rag1tm1Mom (Rag1-/-) mice on the Balb/c background and C57BL/6 mice were obtained from the Jackson laboratories. BAC-transgenic C57BL/6N-Tg (Ccl19-Cre)489Biat (Ccl19-Cre) mice have been previously described. To specifically ablate Bmp4 expression in cardiac fibroblast, we crossed Ccl19-Cre mice with Bmp4fl/fl mice (B6;129S4-Bmp4tm1Jfm/J, obtained from the Jackson Laboratories). Mice were used between 4 and 20 weeks of age depending on the experimental setting. All mice were maintained in individually ventilated cages at 20–24°C and 45–65% ±10% humidity. Food and water available ad libitum.
Wild animals	No wild animals were used in this study
Reporting on sex	Male and female animals were used in all experiments
Field-collected samples	No field-collected samples were used in this study
Ethics oversight	Experiments were performed in accordance with federal and cantonal guidelines (Tierschutzgesetz) under permission numbers SG02/19, SG07/20, SG25/2020 following review and approval by the St.Gallen Cantonal Veterinary Office

Note that full information on the approval of the study protocol must also be provided in the manuscript.

Clinical data

Policy information about clinical studies
All manuscripts should comply with the ICMJE guidelines for publication of clinical research and a completed CONSORT checklist must be included with all submissions.

Clinical trial registration	(i) Cantonal Ethics Committee Zurich permission 2021-01917; (ii) Ethics Committee of Eastern Switzerland permission 2017-01853, (iii) Ethics committee of the Medical University of Graz permission 32-575 ex 19/20
Study protocol	(i) “Immunopathological pathways underlying myocarditis and inflammatory cardiomyopathy – an exploratory study (ImmpathCarditis)"; (ii) secondary investigation using patient samples collected from the MicroDCM cohort, (iii) Graz Endomyocardial Biopsy Registry.
Data collection	i, ii, iii collection of clinical data and blood samples. i and ii; collection of endomyocardial biopsies.
Outcomes	i, iii, on going collection of samples. ii) see publication Gil-Cruz et al Science 2019.

Flow Cytometry

Plots

Confirm that:

☒ The axis labels state the marker and fluorochrome used (e.g. CD4-FITC).

☒ The axis scales are clearly visible. Include numbers along axes only for bottom left plot of group (a 'group' is an analysis of identical markers).

☒ All plots are contour plots with outliers or pseudocolor plots.

☒ A numerical value for number of cells or percentage (with statistics) is provided.

Methodology

Sample preparation	A description of the sample preparation for flow cytometry and FACS sorting is detailed in the methods section.
Instrument	LSR Fortessa BD Biosciences, FACS Melody BD Biosciences

Software	FACSDiva (BD Biosciences, v8.0.1 and v9.0.1) was used to collect the data and FlowJo software v10.6.2 (Tree Star Inc.) to analyze the data. FACSChorus (BD Biosciences, v1.3) was used to set up cell sorting, and R v.4.0.0 was used to analyze the transcriptomic data.
Cell population abundance	High purity of the post-sort fraction was confirmed by downstream sc/snRNA-seq analysis.
Gating strategy	For all flow cytometric analysis, cells were first gated on FSC/SSC to exclude cell debris following by FSC-A/FSC-H and SSC-A/SSC-H to exclude doublets. Dead cells were excluded from analysis by gating on viability dye negative staining. Gating strategy for identifying cell populations in this study is exemplifying in the Extended data figures.

☒ Tick this box to confirm that a figure exemplifying the gating strategy is provided in the Supplementary Information.
